# Determinants of length of stay after cesarean sections in the Friuli Venezia Giulia Region (North-Eastern Italy), 2005–2015

**DOI:** 10.1038/s41598-020-74161-2

**Published:** 2020-11-06

**Authors:** L. Cegolon, G. Mastrangelo, G. Maso, G. Dal Pozzo, W. C. Heymann, L. Ronfani, F. Barbone

**Affiliations:** 1 Institute for Maternal and Child Health, IRCCS “Burlo Garofolo”, Trieste, Italy; 2 Local Health Unit N. 2 “Marca Trevigiana”, Public Health Department, Via Castellana 2, 31100 Treviso, Italy; 3 Department of Cardio-Thoracic and Vascular Sciences & Public Health, Padua University, Padua, Italy; 4 Obstetrics and Gynecology Unit, Hospital “Villa Salus, Venice, Italy; 5 Florida Department of Health, Sarasota County Health Department, Sarasota, FL USA; 6 Department of Clinical Sciences, College of Medicine, Florida State University, Sarasota, FL USA

**Keywords:** Health care, Health policy

## Abstract

Since Italy has the highest cesarean section (CS) rate (38.1%) among all European countries, the containment of health care costs associated with CS is needed, along with control of length of hospital stay (LOS) following CS. This population based cross-sectional study aims to investigate LoS post CS (overall CS, OCS; planned CS, PCS; urgent/emergency CS, UCS), in Friuli Venezia Giulia (a region of North-Eastern Italy) during 2005–2015, adjusting for a considerable number factors, including various obstetric conditions/complications. Maternal and newborn characteristics (health care setting and timeframe; maternal health factors; child’s size factors; child’s fragility factors; socio-demographic background; obstetric history; obstetric conditions) were used as independent variables. LoS (post OCS, PCS, UCS) was the outcome measure. The statistical analysis was conducted with multivariable linear (LoS expressed as adjusted mean, in days) as well as logistic (adjusted proportion of LoS > 4 days vs. LoS ≤ 4 days, using a 4 day cutoff for early discharge, ED) regression. An important decreasing trend over time in mean LoS and LoS > ED was observed for both PCS and UCS. LoS post CS was shorter with parity and history of CS, whereas it was longer among non-EU mothers. Several obstetric conditions/complications were associated with extended LoS. Whilst eclampsia/pre-eclampsia and preterm gestations (33–36 weeks) were predominantly associated with longer LoS post UCS, for PCS LoS was significantly longer with birthweight 2.0–2.5 kg, multiple birth and increasing maternal age. Strong significant inter-hospital variation remained after adjustment for the major clinical conditions. This study shows that routinely collected administrative data provide useful information for health planning and monitoring, identifying inter-hospital differences that could be targeted by policy interventions aimed at improving the efficiency of obstetric care. The important decreasing trend over time of LoS post CS, coupled with the impact of some socio-demographic and obstetric history factors on LoS, seemingly suggests a positive approach of health care providers of FVG in decision making on hospitalization length post CS. However, the significant role of several obstetric conditions did not influence hospital variation. Inter-hospital variations of LoS could depend on a number of factors, including the capacity to discharge patients into the surrounding non-acute facilities. Further studies are warranted to ascertain whether LoS can be attributed to hospital efficiency rather than the characteristics of the hospital catchment area.

## Introduction

Cesarean section (CS) is an obstetric surgical procedure entailing incision of the woman’s abdomen/uterus to deliver her baby. CS can be planned in advance in case of pathological pregnancy course or if a woman with history of CS declined the option of Trial of Labour (TOLAC)^1^. Alternatively, a primary CS becomes frequently necessary during labour, to protect the health of the mother and/or the newborn^1^.

Modern national health services (NHS) are currently under pressure to meet the evolving needs of a continuously aging population. Hospital and inpatient care constitute the largest proportion of health care expenditures in high-income countries, with childbirth being one of the most frequent reasons of hospital admission^2–4^.

The frequency of CS has been rising worldwide, with subsequent risks of post-operative morbidity, prolonged length of hospital stay after childbirth (LoS) and enhancement of associated health care costs^5–9^.

In five regions of Brazil, the country with the second highest CS rate (55.6%) in the world after the Dominican Republic (56.4%), 36.2% out of the total 984,307 labour admissions during 2015 ended up with a CS^10,11^. Approximately 45% of the total 208.5 million United States Dollars (USD) expenditures associated with hospital admissions for childbirth in Brazil were attributable to CS, with reimbursement from the Brazilian national health service (NHS) being proportionate to LoS^11^.

The increasing rates of CS in several countries are pushing health-care organizations to tackle modifiable factors to reduce not only the number of unnecessary CS and related untoward health outcomes, but also prolonged LoS post CS^8,12–19^.

LoS after childbirth, which varies by country, depends on the indication for each CS, on the respective post-operative complications and on the individual recovery capacity of the woman^20^. Albeit LoS reduction could potentially leave the remaining hospitalization days more service intensive and costly^4^, many high-income countries are increasingly applying early discharge (ED) policies proposed by the American Academy of Pediatrics (AAP) and the American College of Obstetricians and Gynecologists (ACOG): 2 days after a spontaneous vaginal delivery (VD) and 4 days following a cesarean section (CS)^21^. For instance, in Canada during 2015–16, out of 368,676 total inpatient hospital admissions due to childbirth, the average LoS was 2.3 days^22^. LoS at Ottawa hospital during 2012–2016 was 46 h out of 16,023 births, being longer following CS (66 h) than VD (37 h)^22^. LoS post CS has diminished more sharply than LoS after VD in the United States (USA), decreasing by 53.8% following CS (7.8 days in 1970, 6.5 in 1980, 4 days in 1992, 3.6 days in 2006) and by 48.7% for VD (3.9 days in 1970, 3.2 days in 1980, 2.1 days in 1992 and 2.0 days in 2014)^23,24^.

Despite being recognized as an important indicator for efficiency, quality and safety of perinatal/postnatal health care delivery, LoS after CS and associated factors has not been investigated in depth^8,25–28^. A thorough analysis of LoS post CS could be useful to evaluate it as metric of quality and efficiency of postnatal care, supporting the ongoing efforts to reduce postnatal maternal morbidity.

Since Italy has the highest CS rate (38.1%) among all European countries, in addition to reducing the number of redundant CS, the containment of health care costs associated with CS – including LoS—is also needed. We previously conducted a study examining LoS post CS in the Friuli Venezia Giulia region (FVG, North-Eastern Italy) during 2005–2015, contrasting hospital performance with a case mix approach^8^. Using the same database, in the present study we investigated the impact of the outstanding factors on LoS following CS, with the view of providing epidemiological figures potentially useful to support the design and evaluation of obstetric care policies in this Italian region. With respect to the previous study, the present work assesses also the impact of obstetric conditions on LoS post CS, which has never been carried out thus far.

## Methods

The methods have been reported in previous papers^8,13,14^ and are herewith briefly described.

### Study design

This is a population-based cross-sectional study to investigate LoS after CS during 2005–2015 in FVG. The study was approved by the Regional Health Authority of FVG, a regional governmental body issuing anonymized patients’ health data routinely collected by the Italian National Health Service (NHS) to research institutions within the frame of approved protocols/studies, overseeing also that the use of health data complies with the current Italian privacy regulations (D.Lgs 101/2018). Since data analyzed in the present study were anonymized and encrypted, informed consent from study participants to conduct this study was waived.

### The database

Data from the 11 maternity services of FVG during calendar years 2005–2015 were extracted from the Regional Repository, an electronic database anonymously storing administrative information from the Italian NHS. The database we analyzed included information from two sources: the hospital discharge forms (HDF, using the respective ICD-9 codes) and the Certificate of Delivery Care (CEDAP, Italian acronym), a formatted questionnaire collecting clinical and personal information on women and newborns (supplementary material, S1)^8,9,13,14,29,30^.

We used the following ICD-9 codes to retrieve the obstetric conditions associated with each childbirth:

- Polyhydramnions: 657.0;
- Oligohydramnions: 658.0;
- Antepartum hemorrhage/abruptio placentae/placenta previa: 641.(0–1–2–3–8–9);
- Obstructed labour: 660.(0–1–2–3–4–5–6–7–8–9);
- Non reassuring fetal status: 656.3;
- Cord prolapse: 663.0;
- Premature rupture of membranes (PROM): 658.1;
- Eclampsia/pre-eclampsia: 624.(4–5–6–7);
- Rh iso-immunization: 656.1.

The rest of data derived from CEDAP, in which delivery mode is defined as follows:

1. Vaginal delivery (VD) without forceps or vacuum extraction;
2. Planned CS (PCS) or CS for failed induction;
3. CS during labour or urgent CS;
4. Forceps extraction;
5. Vacuum extraction;
6. Other forms of VD.

For the purpose of this study, we used the categories 2 and 3, incorporated into OCS. Category 3 indicates UCS.

The 11 facility centres of FVG were anonymized and coded by alphabetic letter from A to K. A and B are second level maternity units (> 1000 annual births and equipped with a neonatal intensive care unit), whereas the other 9 are first level (< 1000 annual births and/or devoid a neonatal intensive care unit).

**Figure 1:**
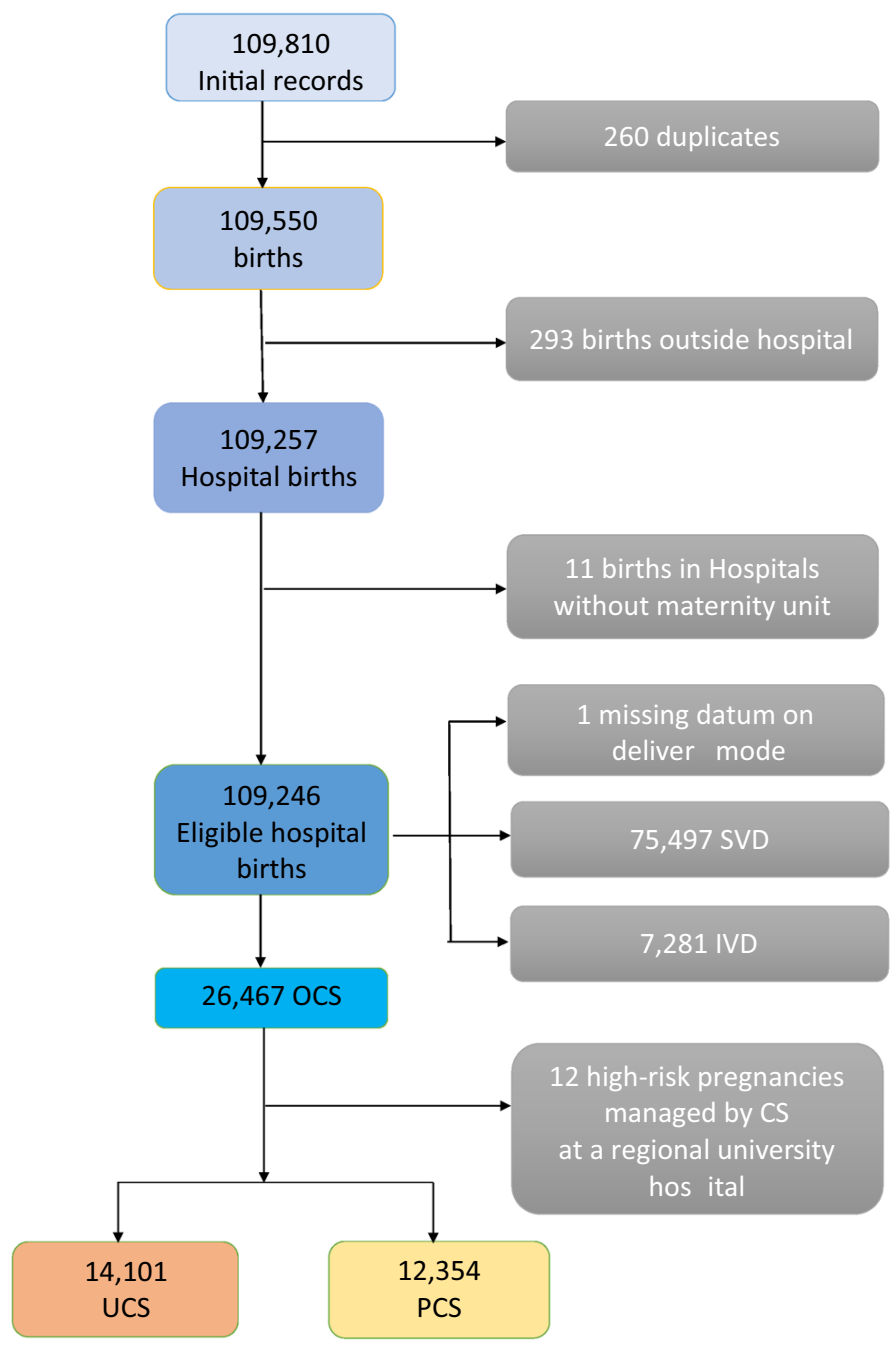
Flowchart displaying the criteria applied to the initial database to obtain the final number of overall cesarean sections (OCS), primary cesarean sections (PCS) and urgent/emergency cesarean sections (UCS). *SVD* spontaneous vaginal deliveries; *IVD *instrumental vaginal deliveries.

Figure 1 shows the flowchart displaying the various criteria applied to the initial database to obtain the final number of hospital births available for the analysis^8^.

### Length of hospital stay after childbirth

LoS (measured in number of whole days) was calculated by subtracting the date of birth by CS from the date of hospital discharge.

As recommended by AAP and ACOG^8–10,21,31^, we considered the average LoS and the percentage of LoS > ED (4 days):

- following overall CS (OCS);
- following planned CS (PCS);
- following urgent/emergency CS (UCS).

**Figure 2:**
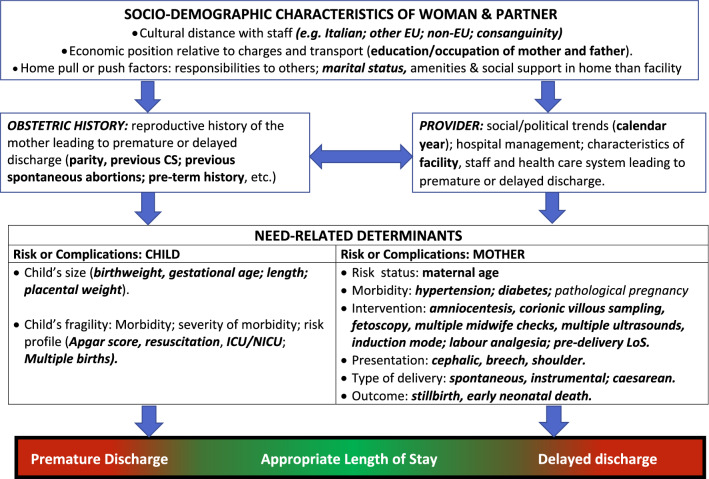
Conceptual Framework explaining the relationship between various factors (not available in our analysis) and length of hospital stay (LoS) after childbirth.

We employed a conceptual framework already proposed, identifying five broad domains of potential determinants of LoS (Fig. 2)^8,9,29^.

1. Health care setting and timeframe (Table 1): hospitals, calendar year, number of births and number of admissions on the delivery day, delivery day of week; seasonality of births;

**Table 1:** Distribution of length of stay after childbirth (LoS, in days) by health care setting and calendar year.

Factors	Strata	All births (N)	OCS			PCS			UCS		
			N (% all births)	LoS (days)		N (% OCS)	LoS (days)		N (% OCS)	LoS (days)	
				M ± SD	> 4 (%)		M ± SD	> 4 (%)		M ± SD	> 4 (%)
Calendar year	2005	10,172	2527 (24.8)	5.0 ± 1.7	58.2	1304 (51.6)	4.9 ± 1.6	56.7	1222 (48.4)	5.0 ± 1.7	59.8
	2006	10,468	2615 (25.0)	4.8 ± 1.5	51.1	1274 (48.7)	4.7 ± 1.4	47.5	1341 (51.3)	4.9 ± 1.7	54.5
	2007	10,648	2700 (25.4)	4.7 ± 1.5	45.7	1300 (48.2)	4.6 ± 1.3	42.8	1396 (51.8)	4.8 ± 1.7	48.4
	2008	10,478	2571 (24.5)	4.8 ± 1.6	46.1	1181 (45.9)	4.5 ± 1.3	40.0	1390 (54.1)	5.0 ± 1.8	51.3
	2009	10,492	2679 (25.5)	4.7 ± 1.7	44.3	1232 (46.0)	4.6 ± 1.6	41.8	1447 (54.0)	4.8 ± 1.8	46.3
	2010	10,406	2547 (24.5)	4.7 ± 1.6	42.7	1204 (47.3)	4.5 ± 1.3	38.0	1343 (52.7)	4.8 ± 1.8	46.8
	2011	9790	2353 (24.0)	4.7 ± 1.8	41.1	1014 (43.1)	4.5 ± 1.5	36.7	1338 (56.9)	4.8 ± 2.0	44.4
	2012	9742	2154 (22.1)	4.6 ± 1.5	37.8	952 (44.2)	4.4 ± 1.3	35.9	1201 (55.8)	4.6 ± 1.7	39.3
	2013	9288	2225 (24.0)	4.6 ± 1.8	36.4	1008 (45.3)	4.4 ± 1.5	33.1	1216 (54.7)	4.8 ± 2.0	39.1
	2014	9092	2090 (23.0)	4.5 ± 1.9	33.8	927 (44.5)	4.4 ± 1.8	31.9	1160 (55.6)	4.6 ± 2.0	35.3
	2015	8658	2006 (23.2)	4.4 ± 1.7	27.9	958 (47.8)	4.2 ± 1.5	23.5	1047 (52.2)	4.6 ± 1.9	32.0
Hospital (Mis a: 193; Mis b: 71)	A	19,059	4430 (23.2)	5.0 ± 1.8	45.9	2016 (45.5)	4.9 ± 1.6	42.8	2414 (54.5)	5.1 ± 1,9	48.4
	B	18,380	6307 (34.3)	4.6 ± 1.6	34.8	3023 (47.9)	4.6 ± 1.5	34.2	3284 (52.1)	4.7 ± 1.8	35.4
	C	8840	1797 (20.1)	4.3 ± 1.2	13.5	936 (52.1)	4.2 ± 1.1	11.7	861 (47.9)	4.3 ± 1.3	15.5
	D	3330	942 (28.3)	5.5 ± 1.4	87.3	351 (37.3)	5.3 ± 1.0	85.4	591 (62.7)	5.6 ± 1.6	88.5
	E	6673	1628 (24.4)	4.7 ± 1.5	31.9	890 (54.7)	4.6 ± 1.4	27.1	738 (45.3)	4.9 ± 1.7	37.8
	F	5723	1469 (25.7)	4.7 ± 1.1	59.9	645 (43.9)	4.6 ± 1.1	57.7	824 (56.1)	4.7 ± 1.1	61.6
	G	9146	1386 (15.2)	4.8 ± 1.3	60.4	544 (39.3)	4.6 ± 1.1	54.7	842 (60.8)	4.9 ± 1.4	64.1
	H	11,681	1920 (16.4)	4.2 ± 1.9	36.0	781 (40.7)	4.1 ± 1.8	30.6	1,139 (59.3)	4.4 ± 1.9	39.7
	I	6047	1303 (21.6)	5.1 ± 1.3	79.6	605 (46.4)	5.0 ± 1.0	79.1	698 (53.6)	5.3 ± 1.5	80.0
	J	12,035	3461 (28.8)	4.3 ± 2.2	25.4	1544 (44.6)	4.0 ± 1.7	22.3	1917 (55.4)	4.5 ± 2.5	27.8
	K	8027	1741 (21.7)	4.8 ± 1.2	68.3	979 (56.2)	4.7 ± 0.9	63.7	762 (43.8)	5.0 ± 1.4	74.1
N. admissions on delivery day (Mis a: 777; Mis b=183)	< 24	23,180	4676 (20.2)	4.7 ± 1.8	41.0	1776 (38.0)	4.5 ± 1.7	38.6	2898 (62.0)	4.8 ± 1.8	42.4
	24–28	31,013	7245 (23.4)	4.7 ± 1.6	42.4	3337 (46.1)	4.5 ± 1.4	39.2	3905 (53.9)	4.8 ± 1.8	45.1
	29–32	25,619	6533 (25.5)	4.7 ± 1.7	43.0	3127 (47.9)	4.5 ± 1.4	39.1	3403 (52.1)	4.8 ± 1.9	46.5
	33 +	28,834	7830 (27.2)	4.7 ± 1.6	44.6	4024 (51.4)	4.6 ± 1.4	41.4	3802 (48.6)	4.8 ± 1.8	47.9
Number of births on delivery day	< 24	22,456	4750 (21.2)	4.7 ± 18	40.5	1848 (38.9)	4.5 ± 1.4	36.6	2889 (61.1)	4.8 ± 1.9	42.9
	24–27	25,644	5885 (23.0)	4.7 ± 1.7	41.7	2662 (45.3)	4.6 ± 1.7	38.6	3220 (54.7)	4.8 ± 1.7	44.2
	28–32	33,123	8408 (25.4)	4.7 ± 1.7	43.7	3991 (47.5)	4.6 ± 1.4	40.6	4412 (52.5)	4.8 ± 1.9	46.5
	33 +	28,011	7424 (26.5)	4.7 ± 1.6	44.6	3853 (51.9)	4.6 ± 1.4	41.3	3570 (48.1)	4.8 ± 1.7	48.1
Delivery day of week	Sunday	13,720	2102 (15.8)	4.8 ± 2.0	45,2	338 (16.1)	4.7 ± 1.6	44.1	1764 (83.9)	4.8 ± 2.0	45.4
	Monday	15,716	3942 (25.1)	4.6 ± 1.5	40.4	1866 (47.3)	4.5 ± 1.3	38.1	2076 (52.7)	4.8 ± 1.8	42.4
	Tuesday	16,979	4638 (27.3)	4.7 ± 1.7	40.6	2541 (54.8)	4.6 ± 1.6	37.5	2097 (45.2)	4.8 ± 1.8	44.5
	Wednesday	16,102	4019 (25.9)	4.7 ± 1.7	44.1	1994 (49.6)	4.6 ± 1.4	41.6	2025 (50.4)	4.8 ± 2.0	46.6
	Thursday	16,446	4497 (27.3)	4.6 ± 1 .7	41.6	2462 (54.8)	4.5 ± 1.6	36.7	2035 (45.3)	4.8 ± 1.8	47.6
	Friday	16,731	4821 (28.8)	4.7 ± 1.4	45.1	2657 (55.1)	4.6 ± 1.3	43.2	2164 (44.9)	4.8 ± 1.6	47.4
	Saturday	13,990	2436 (17.4)	4.8 ± 1.9	45.6	496 (20.4)	4.7 ± 1.5	45.6	1940 (79.6)	4.9 ± 2.0	45.6
Seasonality of births	June-Aug	28,218	6771 (24.0)	4.7 ± 1.6	41.9	3193 (47.2)	4.5 ± 1.4	38.7	3578 (52.8)	4.8 ± 1.8	44.9
	Sep-Nov	28,253	6822 (24.2)	4.7 ± 1.8	41.6	3169 (46.5)	4.5 ± 1.5	37.9	3653 (53.6)	4.8 ± 1.9	44.7
	Dec-Feb	24,658	6558 (24.6)	4.7 ± 1.6	43.3	3097 (47.2)	4.6 ± 1.3	41.4	3461 (52.8)	4.8 ± 1.7	45.0
	Mar-May	26,105	6304 (24.2)	4.7 ± 1.7	45.1	2895 (45.9)	4.6 ± 1.6	41.5	3409 (54.1)	4.8 ± 1.9	48.1
Total		109,234	26,455 (24.2)	4.7 ± 1.7	42.9	12,354 (46.7)	4.5 ± 1.5	39.8	14,101 (53.3)	4.8 ± 1.8	45.7

Mean LoS (M) ± standard deviation (SD); row percentage (%); Mis a: missing values on all births; Mis b: missing values considering only CS.

*CS* cesarean sections, *OCS* overall CS, *PCS* planned CS, *UCS* urgent/emergency CS.
2. Maternal health factors (Table 2): mother’s age, hypertension/diabetes, amniocentesis, villous sampling, fetoscopy, pre-delivery LoS, presentation, labour induction, labour analgesia, neonatal status, number of obstetric checks performed in pregnancy, number of ultrasound (US) scans performed during pregnancy.

**Table 2:** Distribution of Length of Stay after childbirth (LoS, in days) by maternal health factors.

Factors		Strata		All births (N)	OCS			PCS			UCS		
					N (% all births)	LoS (days)		N (% OCS)	LoS (days)		N (% OCS)	LoS (days)	
						M ± SD	> 4 (%)		M ± SD	> 4 (%)		M ± SD	> 4 (%)
Mother age (years) (Mis a: 32; Mis b: 12)		15–19		1254	193 (15.4)	4.9 ± 1.7	48.2	36 (61.0)	4.5 ± 1.1	39.0	23 (39.0)	5.1 ± 1.9	52.3
		20–24		9485	1688 (17.8)	4.8 ± 2.1	43.0	375 (61.7)	4.5 ± 1.4	38.3	233 (38.3)	5.0 ± 2.4	45.7
		25–29		23,674	4879 (20.6)	4.6 ± 1.7	41.5	1272 (61.2)	4.6 ± 1.8	38.8	807 (38.8)	4.7 ± 1.7	43.6
		30–34		38,377	9033 (23.5)	4.6 ± 1.5	42.5	2504 (60.6)	4.5 ± 1.3	39.4	1627 (39.4)	4.7 ± 1.7	45.2
		35–39		28,856	7943 (27.5)	4.7 ± 1.7	43.0	2390 (60.2)	4.6 ± 1.3	39.8	1581 (39.8)	4.9 ± 1.9	46.2
		40–44		7211	2525 (35.0)	4.8 ± 1.7	45.1	762 (58.1)	4.6 ± 1.5	41.9	550 (41.9)	4.9 ± 1.8	48.7
		45 +		345	194 (56.2)	5.3 ± 2.6	60.9	43 (41.8)	5.4 ± 3.2	58.3	60 (58.3)	5.1 ± 1.3	64.0
Hypertension/diabetes (Mis a: 63; Mis b: 14)		No		106,680	25,163 23.6)	4.6 ± 1.6	41.9	11,842 (47.1)	4.5 ± 1.4	39.0	13,321 (82.3)	4.7 ± 1.8	44.5
		Yes		2491	1278 (51.3)	5.6 ± 2.6	63.8	505 (39.5)	5.3 ± 2.4	59.8	773 (60.5)	5.9 ± 2.7	66.5
Villous sampling (Mis a: 6; Mis b:3)		No		104,982	25,204 (24.0)	4.7 ± 1.7	42.8	11,720 (46.5)	4.5 ± 1.5	39.5	13,505 (53.5)	4.8 ± 1.9	45.6
		Yes		4246	1248 (29.4)	4.6 ± 1.4	45.7	47 (51.8)	4.6 ± 1.3	44.8	603 (48.2)	4.7 ± 1.6	46.6
Amniocentesis (Mis a: 6; Mis b:3)		No		91,986	21,367 (23.2)	4.7 ± 1.7	42.4	9790 (45.8)	4.5 ± 1.5	39.5	11,577 (54.2)	4.8 ± 1.9	44.8
		Yes		17,254	5085 (29.5)	4.7 ± 1.6	45.1	2562 (50.4)	4.6 ± 1.4	40.8	2523 (49.6)	4.9 ± 1.7	49.4
Fetoscopy (Mis: 6; Mis b: 3)		No		108,880	26,369 (24.2)	4.7 ± 1.7	42.9	12,309 (46.7)	4.5 ± 1.5	39.8	14,048 (53.3)	4.8 ± 1.8	45.6
		Yes		348	95 (27.3)	4.7 ± 1.4	44.1	43 (45.3)	4.4 ± 1.6	30.2	52 (54.7)	4.9 ± 1.1	56.0
N. obstetric checks (Mis a: 1; Mis b: 0)		< 4		20,851	5587 (26.8)	5.0 ± 1.8	53.2	2721 (48.7)	4.9 ± 1.6	51.3	2866 (51.3)	5.1 ± 2.0	55.0
		4–7		65,797	15,142 (23.0)	4.6 ± 1.7	39.4	7007 (46.3)	4.4 ± 1.4	35.9	8135 (53.7)	4.7 ± 1.8	42.5
		8 +		22,585	5726 25.4)	4.7 ± 1.6	42.1	2626 (45.9)	4.5 ± 1.4	38.4	3100 (54.1)	4.8 ± 1.7	45.3
N. US scans during pregnancy (Mis a: 7; Mis B: 2)		< 4		19,002	3304 (17.4)	4.7 ± 1.8	41.0	1302 (39.4)	4.4 ± 1.5	36.6	2002 (60.6)	4.8 ± 2.0	43.9
		4–5		52,868	11,681 (22.1)	4.6 ± 1.6	40.1	5412 (46.3)	4.4 ± 1.3	36.2	6269 (53.7)	4.7 ± 1.7	43.5
		6 +		37,357	11,468 30.7)	4.8 ± 1.7	46.3	5639 (49.2)	4.7 ± 1.6	44.1	5829 (50.8)	4.9 ± 1.9	48.5
Neonatal status		Liveborn		108,932	26,365 (24.2)	4.7 ± 1.7	42.9	12,330 (46.8)	4.5 ± 1.5	39.8	14,035 (53.2)	4.8 ± 1.8	45.7
		Stillborn		302	90 (29.8)	5.4 ± 3.5	42.2	24 (26.7)	4.5 ± 1.9	37.5	66 (73.3)	5.7 ± 3.8	43.9
Pre-delivery LoS (Mis a: 594; Mis b: 184)		< 3 days		103,757	23,571 (22.7)	4.6 ± 1.5	41.5	10,066 (47.0)	4.5 ± 1.3	38.1	12,505 (53.1)	4.7 ± 1.7	44.6
		3–5 days		3142	1489 (47.4)	5.1 ± 2.2	49.6	628 (42.2)	5.1 ± 2.3	50.6	861 (57.8)	5.1 ± 1.2	48.8
		6 + days		1741	1211 (69.6)	5.7 ± 2.9	61.6	569 (47.0)	5.4 ± 2.4	61.7	642 (53.0)	5.9 ± 3.2	61.5
Any medical assisted fertilization (MAF)	None			108,324	25,895 (23.9)	4.7 ± 1.7	42.6	12,055 (46.6)	4.5 ± 1.4	39.2	13,840 (53.4)	4.8 ± 1.8	45.5
	Drug induced ovulation	N = 80		910	560 (61.5)	5.3 ± 2.0	57.9	299 (54.0)	5.3 ± 1.6	63.6	261 (46.6)	5.3 ± 2.4	51.3
	Intra-uterine insemination (IUI)	N = 181											
	Gamete intra-fallopian transfer (GIFT)	N = 8											
	In vitro fertilization & embryo Transfer	N = 263											
	Intra-cytoplasmic sperm injection (ICSI)	N = 366											
	Other MAF	N = 12											

Mean LoS (M) ± standard deviation (SD); row percentage (row %); M = missing values. Mis a: missing values on all births; Mis b: missing values considering only CS.

*CS* cesarean sections, *OCS* overall CS, *PCS* planned CS, *UCS* urgent/emergency CS.
3. Clinical factors of the child (Table 3), in particular:

**Table 3:** Distribution of length of stay (LoS, in days) after cesarean section (CS) by clinical factors of the child.

Factors		Strata			All births (N)	OCS					PCS				UCS				
						N (% all births)		LoS (days)			N (% OCS)	LoS (days)			N (% OCS)		LoS (days)		
								M ± SD		> 4 (%)		M ± SD	> 4 (%)				M ± SD		> 4 (%)
**Child’s size factors**																			
Gestational age (weeks)		< 29			563	369 (65.5)		5.7 ± 3.1		51.0	47 (12.7)	5.5 ± 3.4	48.9		322 (87.3)		5.7 ± 3.1		51.3
		29–32			1128	853 (75.6)		5.2 ± 2.4		50.4	180 (21.1)	5.2 ± 2.2	55.0		673 (78.7)		5.2 ± 2.4		49.2
		33–36			6213	3215 (51.8)		5.5 ± 2.3		64.8	1155 (35.9)	5.5 ± 2.2	67.1		2060 (64.1)		5.6 ± 2.4		63.5
		37–40			82,631	18,529 (22.4)		4.5 ± 1.4		39.6	10.054 (54.3)	4.4 ± 1.3	36.7		8475 (45.7)		4.6 ± 1.5		43.0
		41 +			18,699	3489 (18.7)		4.5 ± 1.5		37.9	918 (26.3)	4.4 ± 1.2	36.1		2571 (73.7)		4.5 ± 1.5		38.5
Birthweight (g) (Mis a: 5; Mis b: 2)		< 1000			525	328 (62.5)		5.4 ± 2.6		53.7	472 (25.0)	5.3 ± 2.2	54.6		1420 (75.1)		5.5 ± 2.7		52.5
		1000–1499			668	548 (82.0)													
		1500–1999			1328	1016 (76.5)													
		2000–2499			4521	2272 (50.3)		5.5 ± 2.2		67.9	946 (41.6)	5.5 ± 1.9	70.3		1326 (58.4)		5.6 ± 2.4		65.4
		2500–3999			94,947	20,620 (21.7)		4.5 ± 1.4		39.6	10.217 (49.6)	4.4 ± 1.4	44.1		10,403 (50.5)		4.6 ± 1.5		42.6
		4000–4499			6576	1461 (22.2)		4.5 ± 1.5		37.4	719 (43.1)	4.3 ± 1.0	41.2		950 (56.9)		4.7 ± 1.7		41.7
		4500 +			664	208 (31.3)													
Placenta weight (gr) (Mis a: 172; Mis b: 83)		< 500			22,856	5467 (23.9)		5.0 ± 2.0		50.8	2109 (38.5)	4.8 ± 1.6	48.3		3364 (61.5)		5.1 ± 2.2		52.3
		500–599			35,741	6816 (19.1)		4.6 ± 1.4		41.5	3137 (46.0)	4.4 ± 1.3	37.5		3682 (54.0)		4.7 ± 1.5		44.8
		600–999			49,046	12,984 (26.5)		4.5 ± 1.6		38.6	6424 (49.5)	4.4 ± 1.4	35.6		6562 (50.5)		4.6 ± 1.7		41.6
		1000–1500			1420	1106 (77.9)		5.3 ± 2.1		63.1	642 (58.1)	5.2 ± 1.7	64.7		464 (42.0)		5.5 ± 2.5		61.0
Child’s size*		SGA			9122	2929 (32.1)		5.0 ± 1.8		53.4	1298 (44.3)	4.9 ± 1.5	54.0		1631 (55.7)		5.1 ± 2.0		52.9
		AGA			88,127	20,468 (23.2)		4.6 ± 1.7		41.8	9666 (47.2)	4.5 ± 1.5	38.6		10,802 (52.8)		4.8 ± 1.8		44.6
		LGA			11,985	3058 (25.5)		4.6 ± 1.6		40.7	1390 (45.5)	4.5 ± 1.3	35.3		1668 (54.6)		4.8 ± 1.8		45.3
**Child’s fragility factors**																			
Apgar 1 min		<7			6807	2986 (43.9)		5.2 ± 2.5		51.8	771 (25.8)	5.1 ± 2.3	52.0		2217 (74.2)		5.2 ± 2.6		51.8
		7+			102,439	23,469 (22.9)		4.6 ± 1.5		41.8	11,590 (49.4)	4.5 ± 1.4	39.0		11,889 (50.6)		4.7 ± 1.6		44.5
Apgar 5 min		<8			2386	1159 (48.6)		5.3 ± .26		50.9	236 (20.4)	5.2 ± 2.2	53.2		923 (79.6)		5.3 ± 2.7		50.3
		8+			106.860	25,296 (23.7)		4.7 ± 1.6		42.6	12,118 (47.9)	4.5 ± 1.4	39.6		13,178 (52.1)		4.8 ± 1.8		45.3
ICU admission (Mis a: 221; Mis b: 36)		No			103,900	23,243 (22.4)		4.6 ± 1.5		41.3	11,399 (49.0)	4.5 ± 1.4	38.3		11,844 (51.0)		4.7 ± 1.6		44.3
		Yes			5125	3176 (62.0)		5.4 ± 2.5		54.7	932 (29.4)	5.4 ± 2.2	59.8		2244 (70.7)		5.4 ± 2.5		52.7
Resuscitation (Mis a: 54; Mis b: 12)		No			106,764	25,043 (23.5)		4.6 ± 1.6		42.4	12,043 (48.1)	4.5 ± 1.4	39.5		13,000 (51.9)		4.7 ± 1.7		45.0
		Yes			2416	1400 (58.0)		5.4 ± 2.7		53.0	304 (21.7)	5.3 ± 2.4	51.3		1096 (78.3)		5.5 ± 2.8		53.5
Multiple births (Mis a: 898; Mis b: 765)		Singleton		Female	29,603	24,167 (22.7)		4.6 ± 1.6		40.6	11,200 (46.4)	4.4 ± 1.4	36.5		12,967 (53.7)		4.7 ± 1.8		44.1
				Male	31,202														
		Twins or more			1745	1523 (87.3)		5.5 ± 1.9		67.1	784 (51.5)	5.4 ± 1.6	71.6		739 (48.5)		5.5 ± 2.2		62.3

Number (N), row percentage (%); mean LoS (M) ± standard deviation (SD).

*SGA* small for gestational age, *AGA* appropriate for gestational age, *LGA* large for gestational age, *Mis a* missing values on all births, *Mis b* missing values considering only CS, *CS* cesarean sections, *OCS* overall CS, *PCS* planned CS, *UCS* urgent/emergency CS.
4. Child’s size factors: gestational age; birthweight; placenta weight; and a variable “child’s size” created combining the distribution of four factors: sex of child; parity; birthweight and gestational age. The variable “child’s size” enabled to classify newborn into small for gestational age (SGA); appropriate for gestational age (AGA); large for gestational age (LGA)^8,9,13,32,33^.
5. Child’s fragility factors: Apgar score at 1 min; Apgar score at 5 min; resuscitation; intensive care unit (ICU) admission; multiple birth.
6. Socio-demographic background (Table 4), namely: mother’s nationality; marital status of the woman; mother’s education; mother’s occupation; father’s age; father’s education; father’s occupation; consanguinity.

**Table 4:** Distribution of length of stay (LoS, in days) after cesarean section (CS) by socio demographic factors.

Factors	Strata		All births(N)	OCS			PCS			UCS		
				N (% all births)	LoS (days)		N (% OCS)	LoS (days)		N (% OCS)	LoS (days)	
					M ± SD	> 4 (%)		M ± SD	> 4 (%)		M ± SD	> 4 (%)
Father’s age (years) (Mis a: 1949; Mis b: 495)	15–19		199	20 (10.1)	5.1 ± 1.8	55.0	6 (30.0)	4.5 ± 0.8	66.7	14 (70.0)	5.3 ± 2.1	50.0
	20–24		2798	480 (17.2)	4.7 ± 1.7	43.3	153 (31.9)	4.5 ± 1.3	42.1	327 (68.1)	4.8 ± 1.9	43.8
	25–29		12,981	2695 (20.8)	4.7 ± 1.8	41.6	1056 (39.2)	4.6 ± 1.8	37.7	1639 (60.8)	4.7 ± 1.8	44.0
	30–34		31,598	7165 (22.7	4.6 ± 1.6	41.4	3174 (44.3)	4.5 ± 1.3	38.2	3991 (55.7)	4.7 ± 1.8	44.0
	35–39		34,556	8474 (24.5)	4.7 ± 1.6	43.0	4157 (49.1)	4.5 ± 1.3	39.8	4317 (50.9)	4.8 ± 1.9	46.2
	40–44		17,8664	4896 (27.4)	4.7 ± 1.7	43.9	2489 (50.8)	4.6 ± 1.6	41.7	2407 (49.2)	4.8 ± 1.8	46.2
	45–49		5351	1630 (30.5)	4.7 ± 1.5	43.6	797 (48.9)	4.6 ± 1.3	38.7	833 (51.1)	4.8 ± 1.6	48.3
	50–54		1361	420 (30.9)	4.9 ± 1.9	48.1	204 (48.6)	4.6 ± 1.1	43.8	216 (51.4)	5.2 ± 2.4	52.1
	55 +		577	180 (31.2)	4.8 ± 1.6	45.6	99 (55.0)	4.8 ± 1.8	45.5	81 (45.0)	4.7 ± 1.3	45.7
Mother’s nationality (Mis a: 116; Mis b: 36)	EU	Italian	86,083	20,662 (24.0)	4.6 ± 1 .6	42.6	9784 (47.4)	4.5 ± 1.4	39.4	10,878 (52.7)	4.8 ± 1.7	45.5
		Non-Italian	5983	1242 (20.8)	4.4 ± 1.3	35.8	543 (43.7)	4.3 ± 1.2	32.3	699 (56.3)	4.6 ± 1.4	38.5
	Non-EU		17,064	4527 (26.5)	4.9 ± 2.1	46.2	2021 (44.6)	4.7 ± 1.7	43.7	2506 (55.4)	5.1 ± 2.4	48.2
Marital status (Mis a: 8137; Mis b: 2068)	Not married		12,036	2871 (23.9)	4.8 ± 1.8	46.5	1188 (41.4)	4.7 ± 1.7	43.0	1683 (58.6)	4.9 ± 1.8	49.0
	Married		70,340	17,126 (24.4)	4.7 ± 1.7	43.2	8196 (47.9)	4.6 ± 1.4	40.2	8930 (52.1)	4.8 ± 1.9	45.9
	Separated		1136	606 (32.1)	4.7 ± 2.1	43.2	326 (53.8)	4.8 ± 2.4	43.3	280 (46.2)	4.7 ± 1.8	43.2
	Widow		82									
	Divorced		669									
	Living together		16,846	3784 (22.5)	4.6 ± 1.6	40.2	1542 (40.8)	4.5 ± 1.4	38.0	2242 (59.3)	4.7 ± 1.8	41.7
Mother’s education (Mis a: 24; Mis b: 9)	University or more		29,147	6932 (23.8)	4.7 ± 1.6	41.9	3244 (46.8)	4.6 ± 1.5	39.1	3688 (53.2)	4.8 ± 1.7	44.5
	Secondary		52,983	12,612 (23.8)	4.7 ± 1.6	42.7	5791 (45.9)	4.5 ± 1.4	40.1	6822 (54.1)	4.8 ± 1.7	44.9
	Junior secondary		25,103	6343 (25.3)	4.7 ± 1.8	44.0	3052 (48.1)	4.6 ± 1.5	39.7	3291 (51.9)	4.9 ± 2.1	48.1
	Primary/none		1977	559 (28.3)	5.0 ± 2.0	48.1	264 (47.2)	4.9 ± 2.1	45.2	295 (52.8)	5.1 ± 2.0	50.7
Father’s education (Mis a: 6772; Mis b: 1798)	University or more		18,537	4522 (24.4)	4.6 ± 1.6	40.7	2211 (48.9)	4.5 ± 1.4	37.3	2311 (51.1)	4.8 ± 1.7	43.8
	Secondary		51,354	12,154 (23.7)	4.6 ± 1.6	41.4	5605 (46.1)	4.5 ± 1.4	38.7	6549 (53.9)	4.7 ± 1.8	43.7
	Junior secondary		30,762	7505 (24.4)	4.7 ± 1.8	42.8	3509 (46.8)	4.5 ± 1.5	39.4	3996 (53.2)	4.9 ± 2.0	45.7
	Primary/none		1809	476 (26.3)	4.9 ± 2.0	43.1	225 (47.3)	4.7 ± 1.9	39.2	251 (52.7)	5.0 ± 2.1	46.6
Mother’s occupation (Mis a: 448; Mis b: 116)	Unemployed/student/housewife		34,140	8455 (24.8)	4.7 ± 1.9	42.8	3969 (46.9)	4.6 ± 1.7	39.4	4486 (53.1)	4.9 ± 2.1	45.8
	Self-e/entrepreneur		9035	2253 (24.9)	4.6 ± 1.4	41.0	1072 (47.6)	4.5 ± 1.3	37.0	1181 (52.4)	4.7 ± 1.6	44.7
	Manager		2145	579 (27.0)	4.6 ± 1.4	39.7	268 (46.3)	4.5 ± 1.3	36.0	311 (53.7)	4.7 ± 1.5	42.9
	Employed-clerk		30,999	7210 (23.3)	4.7 ± 1.6	43.8	3409 (47.3)	4.6 ± 1.3	41.3	3801 (52.7)	4.8 ± 1.8	46.1
	Blue collar		12,835	3205 (25.0)	4.6 ± 1.5	43.1	1487 (46.4)	4.5 ± 1.3	40.1	1718 (53.6)	4.7 ± 1.6	45.7
	Other (employed)		19,632	4637 (23.6)	4.7 ± 1.6	42.9	2093 (45.1)	4.6 ± 1.6	40.2	2544 (54.9)	4.7 ± 1.6	45.2
Father’s occupation (Mis a: 7145; Mis b: 1922)	Unemployed/student/housewife		3722	1013 (27.2)	4.8 ± 1.8	42.8	461 (45.5)	4.7 ± 1.6	42.0	552 (54.5)	4.8 ± 1.9	43.4
	Self-e/entrepreneur		22,098	5169 (23.4)	4.6 ± 1.6	40.6	2465 (47.7)	4.5 ± 1.3	37.7	2704 (52.3)	4.7 ± 1.8	43.3
	Manager		3337	964 (28.9)	4.6 ± 1.4	37.4	519 (53.8)	4.5 ± 1.3	33.6	445 (46.2)	4.7 ± 1.5	41.8
	Employed-clerk		22,534	5242 (23.3)	4.7 ± 1.6	41.7	2474 (47.2)	4.6 ± 1.4	39.5	2768 (52.8)	4.8 ± 1.8	43.6
	Blue collar		32,809	7985 (24.3)	4.7 ± 1.8	43.0	3695 (46.3)	4.5 ± 1.4	40.0	4290 (53.7)	4.9 ± 2.0	45.7
	Other (employed)		17,589	4160 (23.7)	4.7 ± 1.7	41.6	1878 (45.1)	4.5 ± 1.5	37.2	2282 (54.9)	4.8 ± 1.8	45.2
Consanguinity	No		109,887	26,439 (24.2)	4.7 ± 1.7	42.9	12,339 (46.7)	4.5 ± 1.5	39.8	14,088 (53.3)	4.8 ± 1.8	45.6
	Yes		147	28 (19.1)	4.6 ± 1.3	57.1	15 (53.6)	4.9 ± 1.4	66.7	13 (46.4)	4.3 ± 1.2	46.2

Number (N), row percentage (%); mean LoS (M) ± standard deviation (SD).

*Mis a* missing values on all births, *Mis b* missing values considering only CS, *Self-e* self-employed, *CS* cesarean sections, *OCS* overall CS, *PCS* planned CS, *UCS* urgent/emergency CS.
7. Obstetric history (Table 5): previous livebirths; previous CS; previous stillbirths; previous pre-term births; previous spontaneous abortions; previous neonatal deaths.

**Table 5:** Distribution of length of stay (LoS, in days) after cesarean section (CS) by obstetric history factors.

Factors	Strata	All births (N)	OCS			PCS			UCS		
			N (% all births)	LoS (days)		N (% OCS)	LoS (days)		N (% OCS)	LoS (days)	
				M ± SD	> 4 (%)		M ± SD	> 4 (%)		M ± SD	> 4 (%)
N. previous livebirths	0	58,210	14,516 (24.9)	4.8 ± 1.8	48.7	5383 (37.1)	4.8 ± 1.6	49.2	9133 (62.9)	4.9 ± 1.8	48.4
	1	39,815	9261 (23.3)	4.5 ± 1.5	35.5	5329 (57.5)	4.3 ± 1.3	32.5	3932 (42.5)	4.6 ± 1.7	39.7
	2	8643	2136 (24.7)	4.5 ± 1.6	37.2	1319 (61.8)	4.4 ± 1.3	32.7	817 (38.3)	4.8 ± 1.9	44.4
	3	1820	411 (22.6)	4.7 ± 2.2	37.3	248 (60.3)	4.5 ± 1.6	33.2	163 (39.7)	4.9 ± 3.0	43.6
	4 +	755	131 (17.4)	4.8 ± 2.3	37.7	75 (57.3)	4.5 ± 1.6	28.4	56 (42.8)	5.2 ± 3.0	50.0
N. previous stillbirths	0	108,502	26,137 (24.1)	4.7 ± 1.7	42.9	12,147 (46.5)	4.5 ± 1.5	39.8	13,990 (53.5)	4.8 ± 1.8	45.7
	1 +	744	330 (44.4)	4.7 ± 1.5	41.8	214 (64.9)	4.6 ± 1.3	39.6	116 (35.2)	5.0 ± 1.9	45.7
N. previous cesarean sections	0	100,003	19,556 (19.6)	4.8 ± 1.7	47.4	7731 (39.5)	4.7 ± 1.6	46.8	11,825 (60.5)	4.9 ± 1.8	47.7
	1	8097	5792 (71.6)	4.3 ± 1.4	30.3	3746 (64.7)	4.2 ± 1.1	28.1	2046 (35.3)	4.5 ± 1.8	34.4
	2 +	1146	1107 (96/7)	4.4 ± 1.4	30.6	877 (79.2)	4.3 ± 1.2	28.1	230 (20.8)	4.6 ± 1.9	40.2
N. previous pre-term babies (Mis a:1,144; Mis b: 258)	0	105,764	25,355 (24.0)	4.7 ± 1.7	42.9	11,793 (46.5)	4.5 ± 1.5	39.8	13,562 (53.5)	4.8 ± 1.8	45.5
	1	2039	715 (35.1)	4.7 ± 2.0	38.8	394 (55.1)	4.5 ± 1.4	36.8	321 (44.9)	4.9 ± 2.4	41.3
	2 +	287	127 (44.3)	4.7 ± 1.9	37.6	70 (55.1)	4.6 ± 1.9	33.3	57 (44.9)	4.9 ± 1.9	42.9
N. previous intentional abortion	0	100,643	24,284 (24.1)	4.7 ± 1.7	43.0	11,382 (46.9)	4.5 ± 1.4	39.7	12,902 (53.1)	4.8 ± 1.8	46.0
	1	7037	1733 (24.6)	4.7 ± 1.8	41.7	787 (45.4)	4.6 ± 1.9	41.4	946 (54.6)	4.7 ± 1.7	41.9
	2 +	1554	438 (28.2)	4.9 ± 2.0	42.2	185 (42.4)	4.8 ± 1.9	42.1	253 (57.8)	5.0 ± 2.1	42.2
N. previous spontaneous abortions	0	92,684	22,193 (24.0)	4.7 ± 1.7	42.7	10.216 (46.0)	4.6 ± 1.5	39.6	11,977 (54.0)	4.8 ± 1.8	45.3
	1	12,553	3077 (24.5)	4.7 ± 1.6	44.2	1534 (49.9)	4.5 ± 1.4	40.7	1543 (50.2)	4.8 ± 1.8	47.6
	2	2897	804 (27.8)	4.7 ± 1.6	45.5	399 (49.6)	4.5 ± 1.2	41.1	405 (50.4)	4.9 ± 1.9	49.9
	3 +	1099	381 (34.7)	4.7 ± 1.7	40.8	205 (53.8)	4.6 ± 1.5	40.8	176 (46.2)	4.8 ± 1.2	40.8
N. previous neonatal deaths	0	108,911	26,318 (24.2)	4.7 ± 1.7	42.9	12,267 (46.6)	4.5 ± 1.5	39.8	14,051 (53.4)	4.8 ± 1.8	45.7
	1 +	323	137 (42.4)	4.9 ± 1.8	44.1	87 (63.5)	4.8 ± 1.4	44.8	50 (36.5)	5.0 ± 2.4	42.9

Number (N), row percentage (%); mean LoS (M) ± standard deviation (SD).

*Mis a* missing values on all births, *Mis b* missing values considering only CS, *Self-e* self-employed, *CS* cesarean sections, *OCS* overall CS, *PCS* planned CS, *UCS* urgent/emergency CS.
8. Obstetric conditions (Table 6): oligohydramnios; polyhydramnios; eclampsia/pre-eclampsia; placenta previa/ abruptio placenta/ante-partum hemorrhage; non reassuring fetal status; congenital malformations at birth; cord prolapse; PROM; Rh Iso-immunization; obstructed labour; labour analgesia; labour induction; presentation.

**Table 6:** Distribution of length of stay after childbirth (LoS, in days) by obstetric conditions.

Factors	Strata	All births (N)	OCS			PCS			UCS		
			N (% all births)	LoS (days)		N (% OCS)	LoS (days)		N (% OCS)	LoS (days)	
				M ± SD	> 4 (%)		M ± SD	> 4 (%)		M ± SD	> 4 (%)
Oligohydramnios (missing a: 751; missing b: 195)	No	105,879	25,248 (96.1)	4.7 ± 1.7	43.0	11,867 (47.0)	4.5 ± 1.5	39.7	11,869 (53.0)	4.8 ± 1.8	45.9
	Yes	2604	1024 (3.9)	4.7 ± 1.7	40.8	392 (38.3)	4.6 ± 1.3	41.6	632 (61.7)	4.7 ± 1.9	40.4
Polyhydramnios (missing a 751; missing b: 195)	No	108,048	26,030 (24.1)	4.7 ± 1.7	43.0	12,160 (46.7)	4.5 ± 1.5	39.9	13,870 (53.3)	4.8 ± 1.8	45.7
	Yes	435	230 (52.9)	4.7 ± 1.9	39.6	99 (43.0)	4.5 ± 1.4	32.3	131 (57.0)	4.9 ± 2.1	45.0
Eclampsia/pre-eclampsia missing a: 751; Missing b: 195)	No	107,127	25,310 (23.6)	4.6 ± 1.6	41.7	12,001 (47.4)	4.5 ± 1.4	39.1	13,309 (52.6)	4.7 ± 1.7	44.1
	Yes	1368	958 (69.4)	6.4 ± 3.0	75.3	258 (27.2)	6.0 ± 2.8	75.6	692 (72.8)	6.5 ± 3.1	75.1
Placenta previa/abruptio placenta/ante-partum haemorrhage (missing a: 751; missing b: 195)	No	107,202	25,137 (23.5)	4.7 ± 1.7	42.6	11,832 (47.5)	4.5 ± 1.5	39.5	13,205 (52.5)	4.8 ± 1.8	45.3
	Yes	1281	1123 (87.7)	5.0 ± 2.0	50.5	327 (29.1)	4.9 ± 1.6	50.5	796 (70.9)	5.1 ± 2.1	50.5
Non reassuring fetal status (missing a: 751; missing b: 195)	No	105,786	24,510 (23.2)	4.7 ± 1.7	42.6	12,079 (49.3)	4.6 ± 1.5	39.6	12,431 (50.7)	4.8 ± 1.8	45.5
	Yes	2697	1750 (64.7)	4.7 ± 1.7	46.9	180 (10.3)	4.8 ± 1.6	51.7	1570 (89.7)	4.7 ± 1.7	46.4
Congenital malformations at birth (missing a: 70; missing b: 15)	No	107,644	25,928 (24.1)	4.7 ± 1.7	42.9	12,126 (46.2)	4.5 ± 1.5	39.8	13,802 (52.2)	4.8 ± 1.8	45.7
	Yes	1520	512 (33.7)	4.7 ± 1.8	43.1	220 (43.0)	4.6 ± 1.6	42.0	292 (57.0)	4.8 ± 1.9	43.8
Cord prolapse (missing a 751; missing b: 195)	No	108,410	26,193 (24.2)	4.7 ± 1.7	42.9	12,258 (46.8)	4.5 ± 1.5	39.8	13,935 (53.2)	4.8 ± 1.8	45.6
	Yes	73	67 (91.8)	4.9 ± 2.1	47.8	1 (1.5)	0	0	66 (98.5)	4.9 ± 2.2	48.5
PROM (missing a: 751; missing b: 195)	No	95,699	23,239 (24.3)	4.7 ± 1.7	43.2	11,736 (50.5)	4.5 ± 1.5	39.7	11,503 (49.5)	4.8 ± 1.8	46.7
	Yes	12,796	3021 (23.6)	4.6 ± 1.7	41.2	523 (17.3)	4.6 ± 1.5	42.3	2498 (82.7)	4.7 ± 1.8	40.9
Rh Iso-immunization (missing a: 751; missing b: 195)	No	108,399	26,218 (24.2)	4.7 ± 1.7	42.9	12,242 (46.7)	4.5 ± 1.5	39.7	13,983 (53.3)	4.8 ± 8	45.6
	Yes	96	42 (43.8)	5.0 ± 1.4	59.5	24 (57.1)	4.8 ± 1.5	39.8	18 (42.9)	5.1 ± 1.3	61.1
Obstructed labour (missing 751; missing b: 195)	No	105,056	24,211 (23.1)	4.7 ± 1.7	42.9	11,999 (49.6)	4.5 ± 1.5	39.6	12,212 (50.4)	4.8 ± 1.9	46.2
	Yes	3472	2049 (59.8)	4.7 ± 1.5	43.1	260 (12.7)	4.8 ± 1.6	48.9	1789 (87.3)	4.6 ± 1.4	42.0
Labour analgesia (missing a: 184; missing b:127)	No	89,525	23,100 (87.7)	4.7 ± 1.7	43.5	11,870 (51.4)	4.6 ± 1.5	40.0	11,230 (48.6)	4.8 ± 1.9	47.2
	Yes	19,525	3228 (12.3)	4.6 ± 1.5	39.5	392 (12.1)	4.6 ± 1.6	38.9	2836 (87.9)	4.6 ± 1.4	39.6
Labour mode (missing a: 276 missing b: 36)	Spontaneous	69,481	5962 (22.6)	4.7 ± 1.6	43.6	470 (7.9)	4.5 ± 1.5	42.0	5492 (92.1)	4.7 ± 1.6	43.7
	Induced	17,010	3785 (14.3)	4.7 ± 1.6	43.2	949 (25.1)	4.6 ± 1.4	44.1	2836 (74.9)	4.7 ± 1.6	42.9
	Augmented	6786	991 (3.8)	4.5 ± 1.9	36.2	85 (8.6)	4.6 ± 1.5	42.4	906 (91.4)	4.5 ± 2.0	35.6
	No labour	15,681	15,681 (59.4)	4.7 ± 1.7	43.0	10,821 (69.0)	4.5 ± 1.5	39.3	4860 (31.0)	5.1 ± 2.1	51.2
Placental secondment (missing a: 68; missing b: 15)	Spontaneous	81,859	670 (0.8)	4.4 ± 1.6	39.8	352 (52.4)	4.1 ± 1.5	34.0	318 (47.3)	4.6 ± 1.6	46.2
	Manual/instrumental	27,307	25,770 (97.4)	4.7 ± 1.7	43.0	11,996 (46.6)	4.6 ± 1.5	40.0	13.774 (53.5)	4.8 ± 1.8	45.6
Presentation (missing a: 181; missing b: 164)	Cefalic	103,952	21,272 (20.5)	4.7 ± 1.7	41.7	9276 (43.6)	4.5 ± 1.5	38.2	12,003 (56.4)	4.8 ± 1.8	44.4
	Breech	5288	4893 (92.5)	4.7 ± 1.6	47.4	2945 (60.2)	4.6 ± 1.3	44.0	1948 (39.8)	5.0 ± 1.9	52.5
	Shoulder	126	126 (100)	5.3 ± 2.4	59.2	63 (50.0)	4.8 ± 1.3	53.2	63 (50.0)	5.8 ± 3.0	65.1

Number (N), row percentage (%); mean LoS (M) ± standard deviation (SD).

*Mis a* missing values on all births, *Mis b* missing values considering only CS, *CS* cesarean sections, *OCS* overall CS, *PCS* planned CS, *UCS* urgent/emergency CS.

### Statistical analysis

The mean LoS and the percentage of LoS longer than the proposed ED benchmark following CS (4 days) were calculated for each of the above explanatory factors. The mean LoS and the 0/1 variable LoS (lower/higher than ED) were used as outcomes in a multiple logistic and in a multiple linear regression models, respectively (see below).

Some factors were deliberately dropped from the final multivariate logistic and linear regression model for the following different reasons:

- Apgar score at 1 min and resuscitation due to collinearity with Apgar score at 5 min and intensive care unit (ICU) admission respectively, which both had stronger effect size and we thought they were more plausible to be retained in the final model;
- child's size, due to collinearity with birthweight and gestational age, both with stronger effect size;
- previous spontaneous abortions, as the relative effect was not consistent across the two types of CS;
- father's education, father's occupation, marital status and pre-term history, since in addition of being affected by a large number of missing values, their significance was inconsistent across the two CS types and their effect size was negligible.

**Figure 3:**
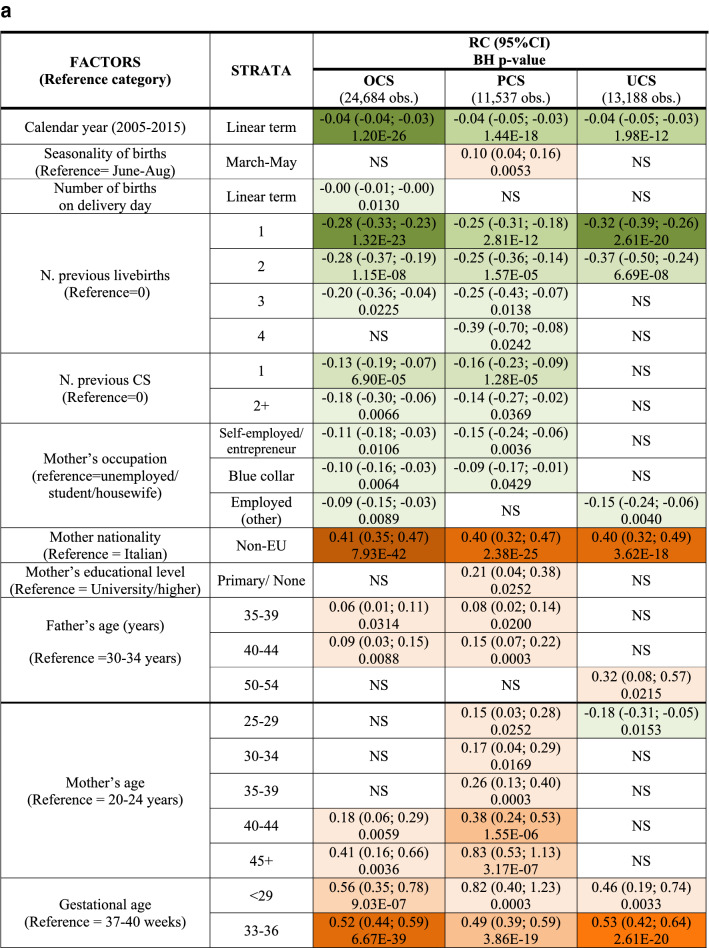

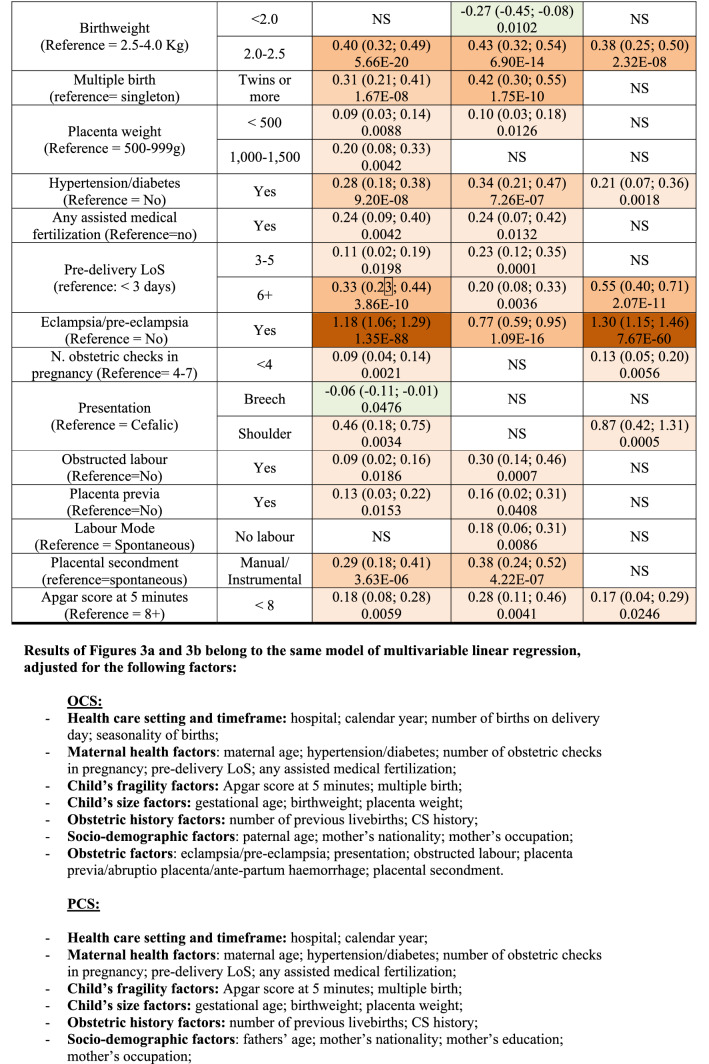

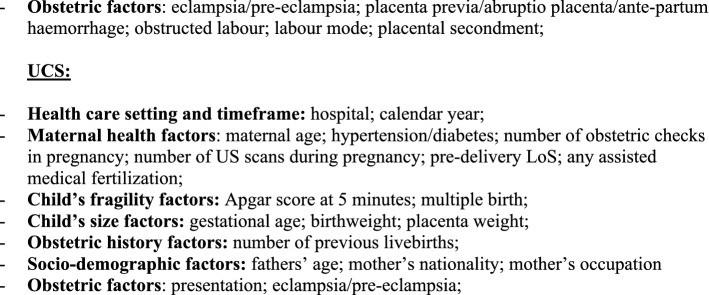

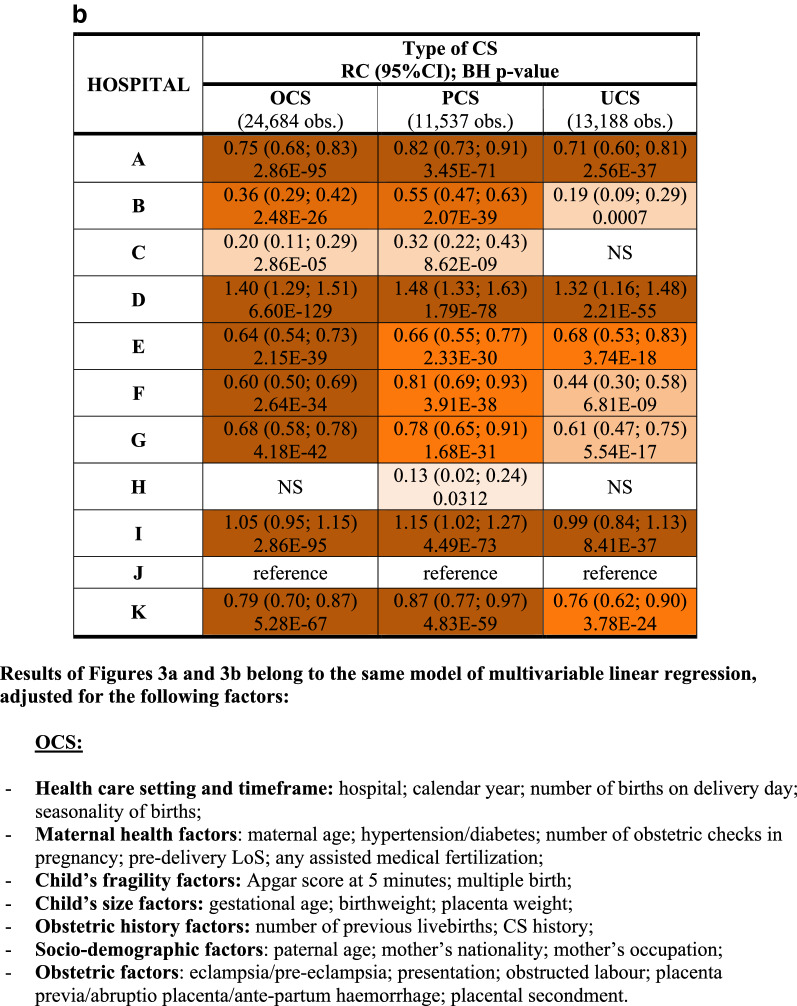

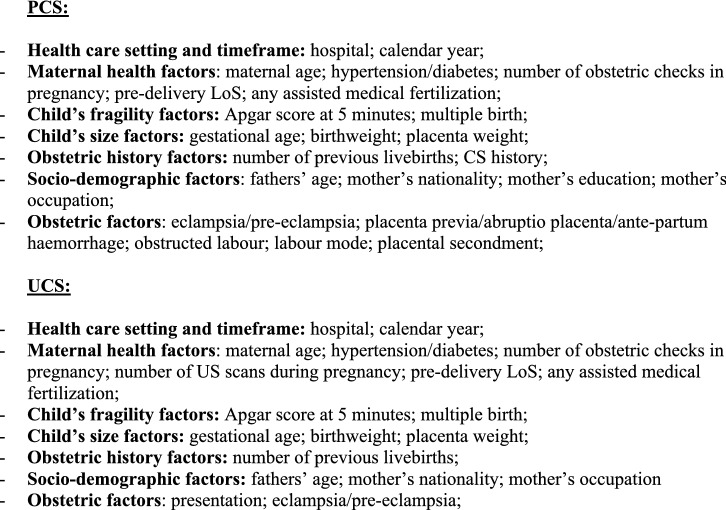
** (a)** Multivariable linear regression model for length of stay (linear endpoint) following overall cesarean sections (OCS), planned cesarean sections (PCS) and urgent/emergency caesarean sections (UCS). Regression coefficients (RC) with 95% confidence interval (95% CI); Benjamini Hochberg (BH) p-value set at 5% discovery rate (bottom of each cell). *obs.*  complete case (analysis) observations. **(b)** Multivariable linear regression model for length of stay (linear endpoint) following overall cesarean sections (OCS), planned cesarean sections (PCS) and urgent/emergency caesarean sections (UCS). Adjusted hospital estimates (regression coefficients, RC), with 95% confidence interval (95% CI); Benjamini Hochberg (BH) p-value set at 5% discovery rate (bottom of each cell). *obs.*  complete case (analysis) observations.

We fitted a multiple linear regression model for each CS type (OCS, PCS as well as UCS), using LoS as a linear endpoint. Stepwise backward selection of independent variables was used to build up all final linear regression models, using p < 0.05 as a criterion. Results were expressed as regression coefficient (RC) with 95% confidence interval (95%CI) and reported in two tables: factors related to mother and newborn health (Fig. 3a); and hospital comparison (Fig. 3b).

**Figure 4:**
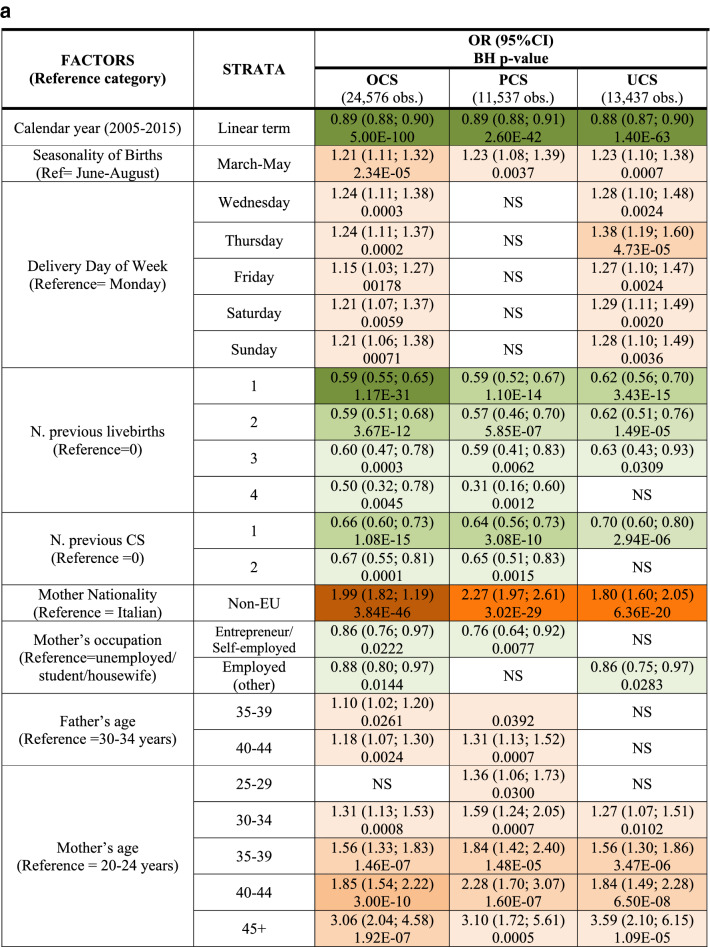

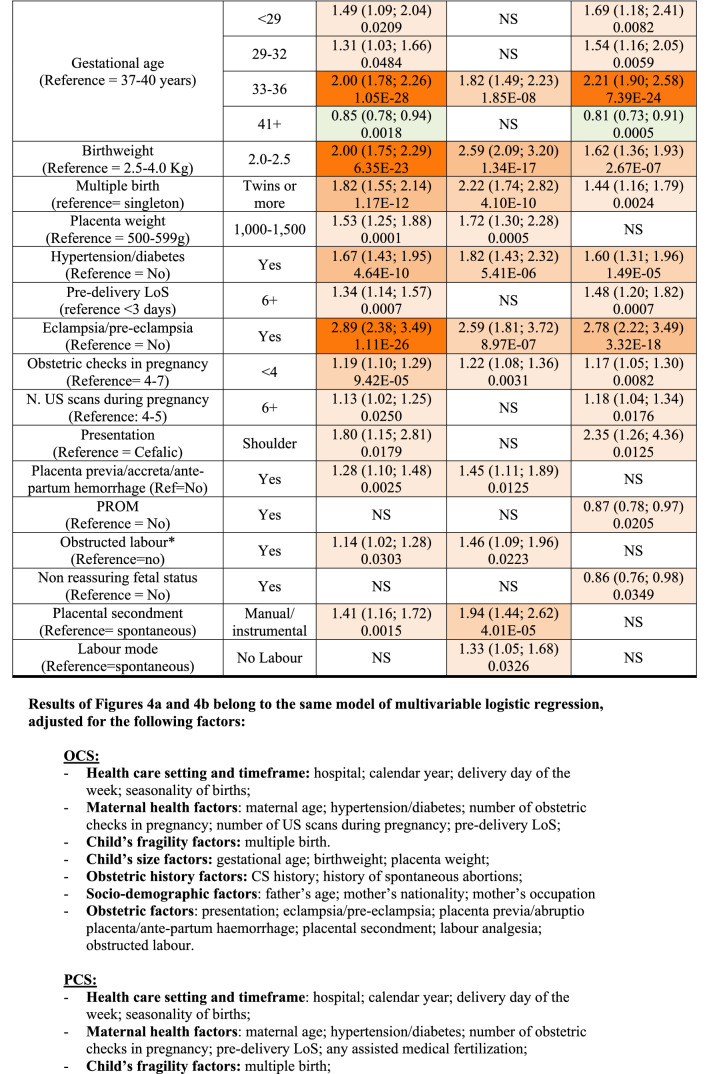

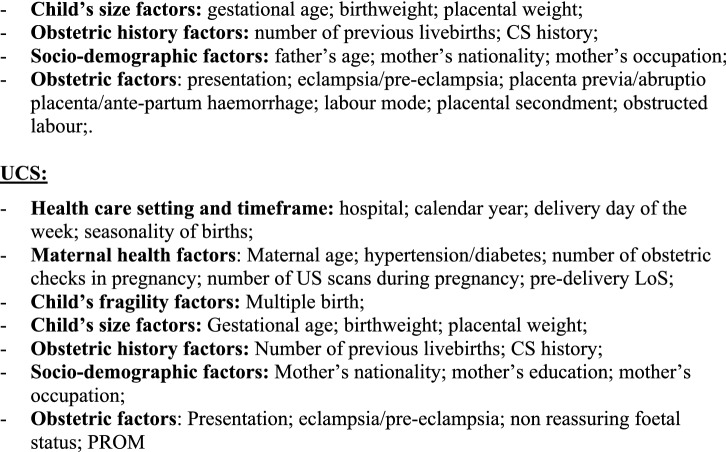

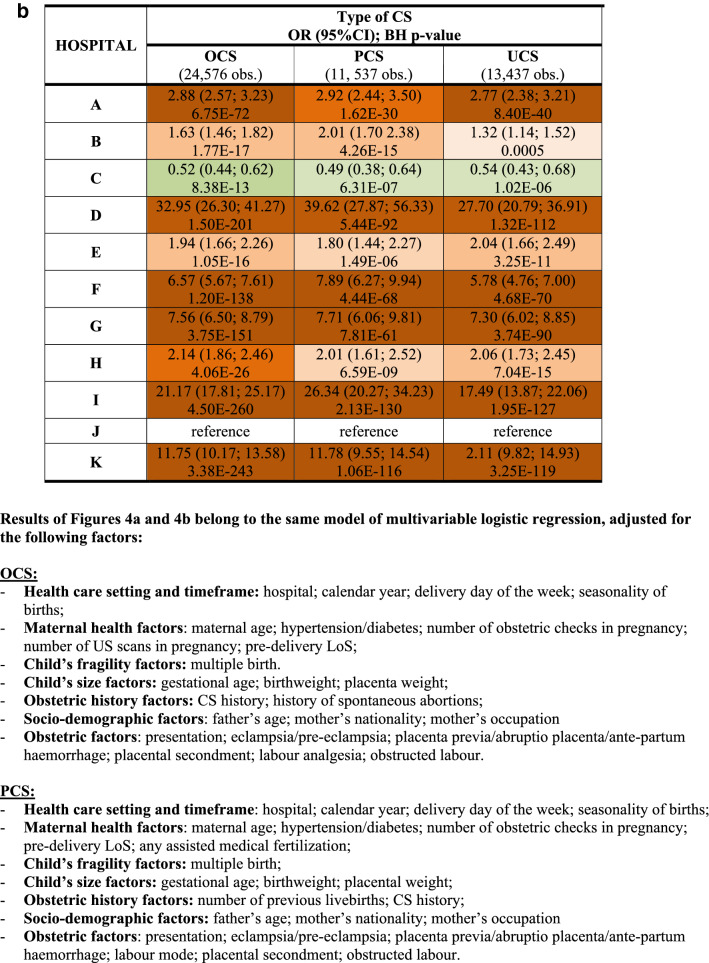

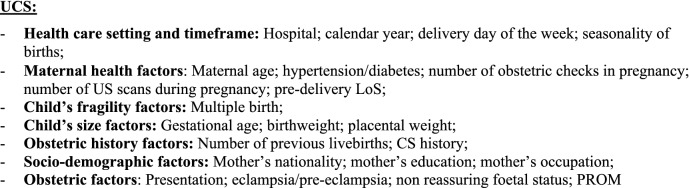
** (a)** Multivariable logistic regression model for length of stay > early discharge (ED, 4 days), following overall cesarean sections (OCS), planned cesarean sections (PCS) and urgent/emergency caesarean sections (UCS). Odds ratios (OR) with 95% confidence interval (95% CI); Benjamini Hochberg (BH) p-value set at 5% discovery rate (bottom of each cell). *obs.*  complete case (analysis) observations. *NS* non-significant. **(b)** Multivariable logistic regression for length of stay > early discharge (ED, 4 days), following overall cesarean section (OCS), planned cesarean sections, and urgent/emergency cesarean section (UCS). Adjusted hospital estimates (odds ratio, OR) with 95% confidence interval (in brackets) and Benjamini–Hochberg p-value, estimated at 5% false discovery rate (bottom of each cell). *NS* non-significant. *Obs*.  complete case (analysis) observations.

Additionally, we fitted a multiple logistic regression model for each CS (OCS, PCS as well as UCS), using LoS as a binary outcome (LoS > ED vs. LoS ≤ ED). Results were expressed as odds ratio (OR) with 95% confidence interval (95%CI) and reported in two tables (Fig. 4a,b) as above.

Results of all regression models (logistic as well as linear) were obtained by comparing each stratum specific estimate (OR and RC) with the corresponding reference category. Hospital J was chosen as reference among all maternity centres, since it was the third maternity centre of FVG in terms of yearly number of births during the entire study period, had the shortest mean LoS after CS among all public hospitals and the second highest CS rate in the region.

Considering the large number of statistical tests performed in the multivariable regression models, some p-values could have been significant by chance. Therefore, we employed as a further selection approach the procedure proposed by Benjamini–Hochberg (BH), setting the false discovery rate at 5% to obtain the BH p-value to be associated with each risk estimate^34^.

Missing values were excluded and complete case analysis was performed. Stata 14.2 (College Station, Texas, USA) was employed for the analysis.

## Results

In the whole FVG during 2005–2015 out of 109,246 hospital births there were a total 24,455 CS occurred in hospitals provided with a maternity unit (24.2% rate), PCS were 12,354 (46.7% out of all CS) and UCS were 14,101 (53.3% of all CS). The mean LoS (and percentage of LoS exceeding the ED threshold of 4 days) was 4.7 days (42.9%), 4.5 days (39.8%) and 4.8 days (45.7%) following OCS, PCS and UCS, respectively.

As can be seen from Table 1, whilst the proportion of LoS > ED decreased for both types of CS over time, the mean LoS post PCS diminished more sharply over the years than UCS. There was considerable inter-hospital variation in the mean LoS after CS, which for PCS varied from 4.0 days in centre J up to 5.3 days in D. The proportion of LoS > ED ranged from 11.7% in centre C to 85.4% in D. Considering UCS, the mean LoS after CS varied from 4.3 days in centre C up to 5.6 days in D. The proportion of LoS > ED for UCS ranged from 15.5% in centre C up to 88.5% in facility D. Examining each hospital separately, the estimates were higher after UCS than PCS, regardless LoS was expressed as mean or percentage > ED. Lastly, increasing mean LoS post CS and LoS > ED were found with higher number of hospital admissions and number of births on delivery day. Always of note from Table 2, for any DM the mean LoS as well as the rates of LoS > ED were smaller on Mondays and Tuesdays, whereas they were higher during winter (December–February) and Spring (March–May) months.

As can be seen from Table 2, across all CS types the mean LoS and the proportion of LoS > ED was particularly higher among women affected by hypertension/diabetes or admitted earlier to hospital (pre-delivery LoS  6+ days). Mean Los and LoS > ED were instead greater among mothers of older age (> 45 years) following PCS, and among stillbirths for UCS. As reported above, larger variations in the outcome measures were found for UCS as compared to PCS and for LoS > ES with respect to mean LoS.

Table 3 shows the distribution of the mean LoS and the proportion of LoS > ED after CS by clinical factors of the child. For all types of CS, both latter outcomes tended to increase with decreasing birthweight and child’s size, with greatest estimates found for low birthweight [LBW (2.0–2.5Kg)]. By contrast, both mean LoS and LoS > ED consistently and considerably decreased as gestational age increased. Regarding placental weight, the outcome estimates were higher at the extremes of the distribution. Always form Table 3, the mean LoS and the proportion of LoS > ED post PCS and UCS tended to be higher in presence of child’s fragility factors: Apgar score at 1 min < 7, Apgar score at 5 min < 8, ICU admission, resuscitation and multiple birth.

As can be seen from Table 4, for both CS types the mean LoS was rather consistent across socio-demographic factors, although it was higher among non-EU women and with lower level of education of both parents. LoS > ED increased progressively with paternal age for both PCS and UCS and was considerably higher among consanguineous parents after PCS.

Table 5 shows the mean LoS and the proportion of LoS > ED after CS by obstetric history factors. Conflicting results between PCS and UCS were found by comparing the mean LoS with the proportion of LoS > ED for obstetric history factors. A reduction of LoS>ED was observed with increasing number of previous livebirths, history of CS and previous pre-term babies. Conversely, the mean LoS tended to increase with history of neonatal deaths and higher number of previous intentional abortions. Less important variations of both outcomes  could be observed in relation with history of stillbirth, spontaneous abortions.

Table 6 shows the distribution of LoS by obstetric conditions/complications. It can be noted that the mean LoS and the proportion of LoS > ED were considerably greater for eclampsia/pre-eclampsia (after both PCS as well as UCS), placenta previa/abruptio placenta/ante-partum haemorrhage (after both PCS and UCS), shoulder presentation (more for UCS) and Rh iso-immunization (following UCS). The proportion of LoS > ED was considerably greater with UCS in case of no labour. For both CS types the mean LoS and the proportion of LoS > ED was remarkably higher for breech and shoulder presentation.

Figure 3a displays the health factors of the mother and the newborn with significant effect on LoS (as a linear endpoint) following OCS, PCS and UCS. LoS significantly decreased over the years, with modest effect size but strong significance, particularly after PCS. Irrespective of the type of CS (PCS or UCS) a longer mean LoS was observed among non-EU women. By contrast, LoS had a clear inverse relation as the number of previous livebirths increased. LoS was also significantly shorter with history of CS only after PCS. An important increase in mean LoS was associated with eclampsia/pre-eclampsia, with much stronger significance following UCS. A greater LoS was observed in older women after PCS, with stronger and clearer trend as mother’s age increased. Furthermore, a strongly significant increase of LoS was found at 33–36 weeks of gestation (irrespective of type of CS). Likewise, LoS was longer only in women with LBW infants (for both PCS and UCS). Multiple births involved a significant increase in LoS following PCS. Moreover, regardless the type of CS, LoS was significantly longer when the mother was affected by hypertension/diabetes, particularly after PCS. Longer pre-delivery LoS as well as numerous obstetric checks during pregnancy had a tendency to increase LoS post-CS, mainly for UCS. Shoulder presentation resulted in longer LoS post UCS, whereas obstructed labour, placenta previa/abruptio placenta/ante-partum haemorrhage and Apgar at 5 min score > 7 were factors associated with longer LoS mainly post PCS.

Figure 3b relates to the differences among maternal centres of FVG. The RCs are expressed in the same unit (days) of the outcome variable (LoS). Both unadjusted and adjusted mean LoS was lower for PCS than UCS cases in all hospitals (Table 1 and Fig. 3b). All centres have a RC higher than the reference centre. Among PCS cases, RC was longer > 1 day in two centres (D, I), between 0.5 and 1 day in five hospitals (K, A, G, E, F in decreasing order of effect size), and < 0.5 day in two maternal units (B, C). Adjusted RCs were comparatively lower among UCS cases. Since they belong to the same model of multivariable linear regression, results of Fig. 3b are adjusted for the same factors displayed at the bottom of Fig. 3a. Therefore, the wide differences among maternity centres cannot be attributed to the case mix.

Unlike Figs. 3a,b, 4a,b use LoS as binary outcome (LoS > ED vs. LoS ≤ ED) instead of linear endpoint. Since the same regression techniques were carried out in all tables, Fig. 4a,b were similar to Fig. 3a,b. Therefore, calendar year, number of previous livebirths and CS history were significantly associated with reduced odds of LoS > ED for both UCS and PCS (Fig. 4a). By contrast, for both CS types LoS > ED was more likely in non-EU mothers, eclampsia/pre-eclampsia, pre-term gestations (33–36 weeks), LBW (2.0–2.5 kg) and hypertension/diabetes. However, whilst the association of LoS > ED with pre-term gestation and with eclampsia/pre-eclampsia was much stronger for UCS, for LBW and mother’s nationality it was stronger following PCS. Other important factors predominantly associated with LoS > ED after PCS were multiple birth and increasing maternal age (Fig. 4a). As can be seen from Fig. 4b, all maternity centres but C were by far more likely to surpass the ED benchmark than the reference (centre J). A similar pattern was observed in the multiple linear regression model (Fig. 3b), although in the latter model centre H was the maternity unit less differing from the reference. The discrepancy can be explained by the criterion “shortest mean LoS post CS among all public hospitals of FVG” used in the choice of hospital J as reference. Hospital C was the only private hospital in FVG.

Interestingly, as can be noted from Fig. 4a, the adjusted OR of LoS > ED was higher than reference (Monday) in all weekdays but Tuesday, with higher degree of significance for Wednesday and Thursday. Further, although with relatively weak significance, for all types of CS LoS > ED was significantly higher during spring months (March–May) than the reference (summer months, June–August).

**Figure 5:**
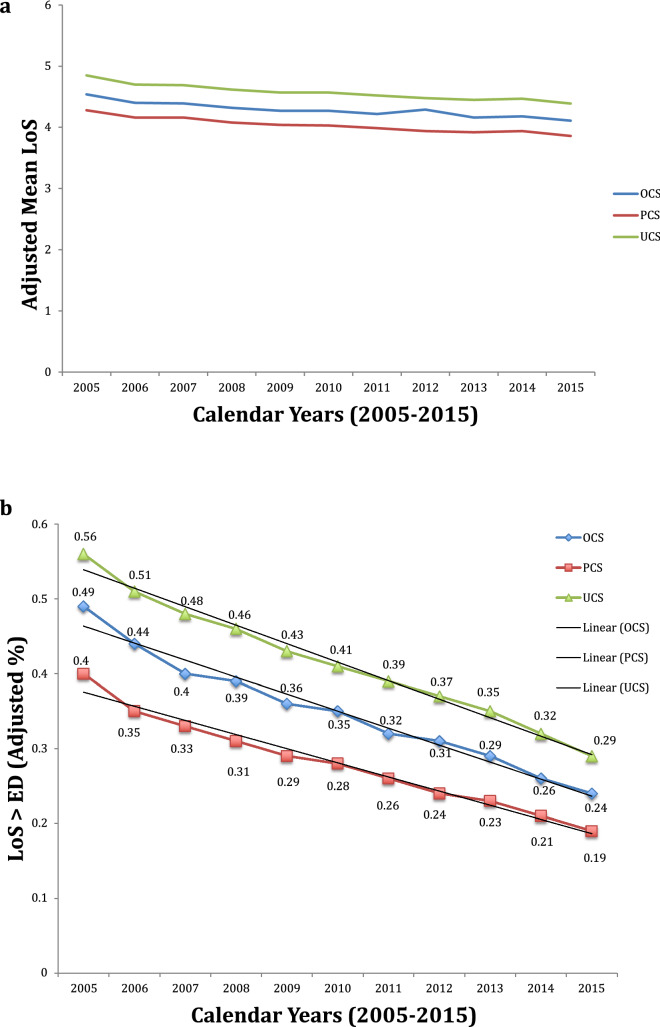
**(a)** (upper panel). Adjusted mean of length of hospital stay (LoS, in days) after after overall cesarean sections (OCS), planned cesarean sections (PCS and urgent/emergency cesarean sections (UCS) over time in Friuli Venezia Giulia (FVG), 2005–2015. Estimates adjusted for the same factors displayed at the bottom of Fig. 3a,b. **(b)** (lower panel). Adjusted proportions of length of hospital stay (LoS) > early discharge (ED) benchmarks (= 4 days) for after overall cesarean sections (OCS), planned cesarean sections (PCS and urgent/emergency cesarean sections (UCS) over time in Friuli Venezia Giulia (FVG), 2005–2015. Estimates adjusted for the same factors displayed at the bottom of Fig. 4a,b.

Figure 5a,b display the mean LoS and the proportion of LoS > ED over time in FVG, adjusted for the same factors included in the above mentioned linear (Fig. 3a,b) as well as logistic (Fig. 4a,b) regression models, respectively. As can be seen, there was a clear decreasing trend of LoS > ED over the years for all three types of CS, whilst the temporal diminishment of the mean LoS was less pronounced.

**Figure 6:**
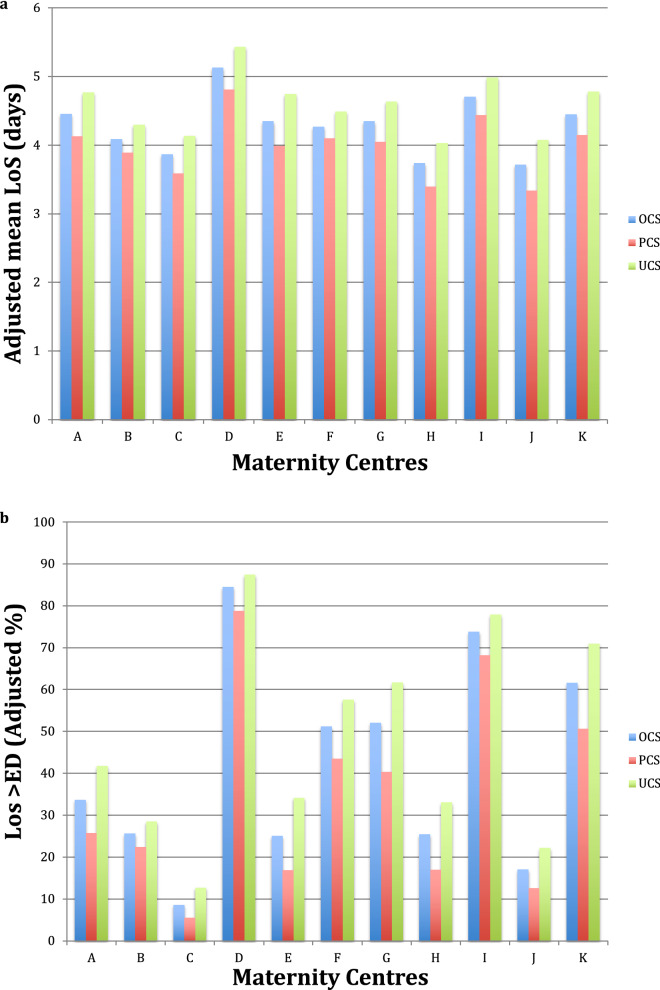
**(a)** (upper panel). Adjusted mean length of hospital stay (LoS) after overall cesarean sections (OCS), planned cesarean sections (PCS) and urgent/emergency cesarean sections (UCS) by maternity centres of Friuli Venezia Giulia (FVG), 2005–2015. Estimates adjusted for the same factors displayed at the bottom of Fig. 3a,b. **(b)** (lower panel). Adjusted rates of length of hospital stay (LoS) > early discharge (ED) benchmarks (= 4 days) after overall cesarean sections (OCS), planned cesarean sections (PCS and urgent/emergency cesarean sections (UCS) by maternity centres of Friuli Venezia Giulia (FVG), 2005–2015. Estimates adjusted for the same factors displayed at the bottom of Fig. 4a,b.

Figure 6a,b display the mean LoS and the proportion of LoS > ED by maternity centres of FVG during the study period, adjusted for the same factors fitted in the above mentioned linear (Fig. 3a,b) as well as logistic (Fig. 4a,b) regression models. A clear adjusted hospital variability can be noted, more pronounced for LoS > ED.

## Discussion

### Key findings

In the entire FVG during 2005–2015, the mean LoS was 4.5 days (39.8% > ED) following PCS and 4.8 days (45.7% > ED) for UCS; a significant decreasing trend over time of LoS > ED was observed for both PCS and UCS. LoS > ED was less likely on Mondays and Tuesdays and more likely during spring months (March–May). With the exception of mother’s nationality (very strong association), prolonged LoS was mainly driven by the clinical conditions of the mother (eclampsia/pre/eclampsia, hypertension/diabetes) and the newborn (gestational age < 36 weeks, birthweight 2.0–2.5Kg). After adjusting for the major medical and obstetric conditions/complications, the strongest determinant of LoS post CS was inter-hospital variation. All maternity centres but C were by far more likely to surpass the ED benchmark than the reference (hospital J). A similar pattern was observed in the multiple linear regression model. These differences could be targeted by policy interventions aimed at their reduction, taking into account the different case mix between hospitals of first and second level.

### Interpretation of findings

LoS is an easily available indicator of hospital activity, being an indirect estimator of resources consumption and efficiency. The hospital variability we found on Los post CS may be due to a number of factors, including differences in practice pattern, service efficiency, discharge policies, experience/ability of obstetric staff and patient/family preferences^35^. Hospitals A and B are referral centres normally managing more complicated and serious obstetric conditions and some women delivering in the latter two centres may live quite far, hence these logistic barriers may push obstetricians to retain women admitted longer. By contrast, LoS was lowest for centres C and J, both located in the same local health unit (LHU) of FVG. The latter LHU provides domiciliary services to puerperae unable to go to hospital for a check-up during the first 10 days following ED for childbirth. These home visits are conducted by community midwives operating in health districts affiliated to the latter LHU.

Decreasing LoS inarguably increases demands on community postnatal services, the quantity and quality of which appears to vary globally^36^. For example, in Iceland, women are offered 8 home visits in the first 10 days postpartum, and their feed-back on postnatal care is generally positive^37^. By contrast, in Australia women are meant to receive at least two weeks postnatal support within their homes but continue to report low satisfaction with postnatal care as compared to antenatal and intrapartum services^38^. In the UK community postnatal care is provided by midwives, and although the National Institute for Health and Care Excellence (NICE) previously recommended a minimum of three home contacts post-childbirth^39^, many women are now asked to attend postnatal clinics instead, and there are no standards regarding the total number of post-partum contacts women should receive^40^. As such, wide variation is found in the number of postnatal contacts experienced by new mothers in the UK. A recent report from the UK National Maternity and Perinatal Audit (NMPA) project team found that the number of planned postnatal contacts for healthy women and babies ranged from 2 to 6, with a median of 3^41^. In an earlier survey of the Royal College of Midwives (RCM), 14% women in the UK reported that they only received one visit and a small minority reported no visit whatsoever^41^.

Interestingly, in the present study LoS > ED was less likely with increasing calendar year and with CS history, whereas it was far more likely among non-EU mothers. This suggests a positive approach of health care providers of FVG in decision making on LoS post CS, with socio-demographic and obstetric history factors probably taken into account.

Non-Italian women may have less family support, therefore may have benefited from longer LoS in FVG for a number of reasons, including inception and adaptation to breast-feeding. However, the impact of nationality and ethnicity may vary by type of health system. In countries adopting the voluntary health insurance (VHI), as the USA, the underlying dynamics on LoS may probably be different. For instance, findings from a secondary analysis of the Maternal–Fetal Medicine Units Cesarean Registry on 26,000 low-risk American women with singleton pregnancies, liveborn at 24–40 weeks, known ethnicity, up to 2 prior CS, and scheduled obstetric surgical procedures concluded possible disparities in quality and efficiency of obstetric care delivered to minorities^49^. Non-Hispanic Black women were more likely to incur longer LoS in the latter study, even after stratification by gestational age and type of CS, whereas Hispanic mothers had significantly shorter LoS across all gestational ages^42^. In another postnatal survey in 19 USA states during 2000, using data from the Pregnancy Risk Assessment Monitoring System, ED was more likely among Hispanic and Black women^43^. Lastly, in another population-based postnatal survey conducted in 1999 on 2828 Californian women with low risk singleton pregnancies, ED was associated with lower socio-economic status, with untimely follow up more likely among latinas and non-English speaking women^44^.

Following inter-hospital variability, calendar year, number of previous livebirths and nationality of the woman, in the present study prolonged LoS after CS was influenced by child size factors. In particular we found LBW (2.0–2.5 kg) and pre-term gestations (33–36 weeks) both being strong determinants of prolonged LoS after PCS as well as UCS. A huge fraction of overall neonatal costs are reportedly leveraged by LBW and/or premature babies^45^, accounting for half newborn hospitalizations and 25% pediatrics costs in the USA^46^. In addition to decrease mortality/morbidity, interventions to delay or prevent premature deliveries could have a major impact on the containment of pediatric and newborn expenditures^46,47^. In a California study on 518,704 deliveries from the 2000 birth cohort, total adjusted hospital costs and LoS were calculated for both mothers and infants^45^. Total hospital costs for mothers comprised adjusted inpatient costs for any antenatal admissions as well as for postpartum hospitalizations, whereas for newborns they included adjusted inpatient costs associated with childbirth and with following hospital accesses (transfers or re-admissions) prior to primary discharge or before death, in case of child’s decease before discharge. Whilst newborns weighing > 2500 g at birth had a mean LoS of 2.3 days, the respective estimate for LBW infants varied extensively from 6.2 to 68.1 days^45^. Newborns affected by very low birthweight (VLBW) burdened 0.9% deliveries but 35.7% total hospital costs, whereas LBW infants accounted for 5.9% births but 56.6% costs. We did not have information on hospital costs associated with childbirth, also because lack of information on sensitive data prevented the follow-up of infants across hospital registries. However, LBW accounted for 4.1% of all births and 8.6% OCS, whereas VLBW were 2.2% out of all deliveries and 7.2% of all OCS in the present study.

Various pre-existing obstetric conditions as well as potentially preventable peri-surgical complications are associated with extended LoS post CS according to the open literature, including labour induction, labour augmentation (by oxytocin administration), ruptured membranes > 24 h, and epidural analgesia^48–50^.

Although we did not find any association with labour analgesia, there is evidence that the type of anesthetic technique employed is a strong predictor of extended LoS after CS, with longer hospitalization found with administration of epidural than spinal analgesia^12^. A study investigated 1,619 women undergoing CS during 2002–2005 at Aretaieio Hospital (Athens, Greece) in relation to the type of anesthesia administered. Although the impact of general anesthesia on LoS post CS decreased over the years in the latter study, neuraxial anaesthesia for CS was associated with shorter LoS than general anesthesia, and it was also influenced by the skill/ability of the surgeon^51^. A study at Ochsner clinic in New Orleans (Louisiana, USA) examined 840 consecutive parturients over a 1-year period. Prolonged LoS after CS was observed in 14.3% deliveries and was influenced by the type of anesthetic approach employed and the amount of intraoperative fluids administered during CS^12^. Among 57,812 women undergoing CS in USA between 1999 and 2002, within the network of the National Institute of Child Health and Human Development, independent obstetric risk factors for prolonged LoS included peri-surgical morbidities (general anesthesia, uterine atony, transfusion, hysterectomy, endometritis, ileus, wound and hemorrhage related complications), and perinatal conditions (pre-term gestation, birthweight). The most significant factors associated with extended LoS were ileus, endometritis and wound complications, but not general anesthesia^52^. In the present study we cannot fully address the latter question, since until 2015 CEDAP data did not include details on the type of analgesia administered.

Hypertension/diabetes, pre-delivery LoS > 5 days and < 4 obstetric checks in pregnancy were equally associated with longer LoS post both PCS and UCS. Hypertension and eclampsia were factors significantly associated with longer LoS post CS also in the above study on 840 women undergoing CS at Ochsner clinic in New Orleans ^12^. Pre-eclampsia and severe eclampsia (along with decreased gestational age, vaginal bleeding in the second half of pregnancy and suspected intrauterine growth retardation) are recognized prenatal factors associated with extended LoS^48–50^. The clinical conditions of the woman during pregnancy, including also pre-existing medical disorders (e.g. cardiovascular, respiratory, infectious, neurologic, autoimmune disease, etc.—factors not considered in our study) seemingly influence also the risk of readmission. For instance, in a USA study using the Healthcare Cost and Utilization Project's (HCUP) Nationwide Readmissions Database on 65,401 women affected by pre-eclampsia undergoing CS during 2014, 1016 (1.6%) had to be readmitted for hypertensive disorder and 90.6% of these readmissions occurred during the first 10 days following discharge. In the latter study longer LoS (> 5 days) was associated with lower adjusted risk of readmissions for hypertensive disorders within 60 days after discharge. Postpartum care is critical in determining the subsequent risk of readmission for sequelae related to eclampsia and pre-eclampsia, hence longer LoS following CS may be recommended in these conditions^53^. We could not fully confirm such findings related to readmission because of confidentiality of sensitive patients’ data.

Although with minor level significance, LoS > ED was more likely in all days but Tuesday, with higher level of significance for Wednesday and Thursday. In Italy the civil registration offices are closed on Saturday and Sunday, therefore despite women delivering on Wednesday, Thursday or Friday may potentially be eligible to be discharged over the week-end, they are retained in hospital until Monday, when the will be able to register their child at the city council. By contrast, women delivering on Monday or Tuesday are more likely to be discharged by Friday.

CS performed during spring months (March–May) were associated with LoS > ED for both PCS, UCS and PCS, whereas the adjusted mean LoS during these 3 months was significantly higher only for PCS. Although with relatively weak significance, these findings slightly deviate from a previous study reporting higher risk of prolonged LoS post VD during winter (December–February) as well as spring months (March–May)^9^. The impact of cold weather and related morbidity would in fact be expected to be higher during winter months, where temperatures are usually lower and the risk of respiratory infections (especially influenza) higher. However, despite being lower than spring months, the crude rate of LoS > ED was still higher during winter months as compared to summer and autumn months for all three types of CS (OCS, PCS and UCS). Moreover, within spring months there was a declining trend of LoS > ED for OCS from March (46.8%), to April (44.7%) and May (43.6%). For PCS, the respective rates were 44.0%, 41.3% and 39.0%; for UCS they were 49.3%, 47.4% and 47.6% respectively. The latter figures suggest a decreasing effect over time of weather temperature and influenza risk on LoS post CS.

### Hospital costs

Italy, which offers universal health coverage, is among the growing number of countries adopting a prospective payment system based upon capitation grants and diagnosis-related groups (DRGs), which fix the payments by estimated costs of hospital care ahead of service delivery. The DRG system has the advantage of stimulating the provider to contain the cost for each medical service, including unnecessary days of prolonged LoS^4^. The contingency capacity, bed turn-over and rationalization of available resources may have different impact on various hospitals. Nevertheless, in all multivariable models LoS was not influenced by number of admissions and number of births on delivery day at regional level, suggesting no impact of bed turnover on LoS. Governmental investments should be allocated to encourage measurements and controls of such differences, in order to maintain equity of health outcomes and costs across maternity services.

### Prospects

The desirable model of obstetric care should be patient-centered and should deliver high quality of medical services yet containing health care costs by minimizing unnecessary prolonged LoS. Various models of postnatal management have been studied, delivering home-based, outpatient or inpatient services. These models consider and pursue different endpoints, including patient satisfaction, breastfeeding rates, health care costs and hospital readmissions for both women and newborns^54–57^. Integrated programs of primary and secondary care services, entailing frequent follow up home visits post hospital discharge (conducted by community midwives, nurses and/or general practitioners) seem capable of diminishing hospital re-admissions whilst ensuring quality of care and patient satisfaction. Nonetheless, these models of care may not be accessible and deliverable in every community setting, since they may be demanding in terms of organizational and human resources^57^. As a result, since it depends on the capacity to discharge patients into the surrounding non-acute facilities, LoS could become a debatable indicator of hospital ‘efficiency’, as its variation could be explained by the characteristics of the hospital catchment area^58^.

An interventional community-based outpatient postnatal clinic, the Monarch centre, was set up at Ottawa hospital (Canada) during 2014, with the aim to provide coordination between hospital care, community and primary care services. Pre-booked appointments were scheduled within 48 h of hospital discharge following childbirth. A number of services were provided, including mood screening/management, neonatal care, laboratory testing, breast-feeding assessment and support. General practitioners, lactation consultants and registered nurses were available for consultation on appointment. Out of 16,023 deliveries occurring between January 2012 to December 2016, the mean LoS was 46 h (66 h after CS vs. 37 h post VD). Eighteen months after the intervention, the average LoS for CS decreased by 20 h (significantly reducing by 27%); LoS post VD instead decreased much less (6 h), by 18%, but it was still significantly. Readmission rates of neonates at 30 days post discharge just rose from 1.1 to 1.9%^22^. Therefore, the implementation of integrated primary and secondary care services seems the key approach to contain unnecessary prolonged LoS after CS.

## Strengths and limitations

Strengths of this study have been outlined elsewhere^8,9^.

Because for years 2005–2015 the CEDAP questionnaire collected time of childbirth but not hospital discharge’s, we had to use day metrics instead of hours to estimate LoS. Although this is an important limitation, as differences in hours of LoS may have an impact on wellness of the woman and her family, the calculation of hospital costs by LoS in Italy is based upon days. However, in future it would be important for CEDAP to accurately record information on time of admission, time of birth and time of discharge.

As explained above, we did not have information on the address of residence of the woman, a logistic aspect that may have a major influence on decision making on LoS if the new mother lives far from the respective delivery facility. This is particularly the case for the two referral centres, which presumably receive more women from distant locations of FVG or even outside. It would therefore be very important in the future also to take into account the council of residence of the woman, in compliance with the Italian privacy law.

Although labour induction was limited to 15.6% out all deliveries in our studies, in the future it would be important for CEDAP to distinguish PCS from CS for failed induction. Moreover, UCS should be separated from CS during labour.

In the future it would be important for CEDAP also to collect information on other factors that may have an impact on LoS: type of analgesia administered during delivery; smoking status; body mass index (BMI); physical activity; amount of bleeding during delivery; confidence of the mother with breastfeeding and her readiness for discharge,

Finally, although our database had a high level of completeness and accuracy of data, some important socio-demographic information (as father’s education, father’s occupation and marital status) were affected by a relevant number of missing values. Although this may reflect the woman’s reluctance to reveal some personal (though anonymous) information, in the future it would be important to further improve the completeness of data collection by CEDAP, abandoning any form of paper document in favor of a standardized regional software for real time check-up of data entry, preventing input of inconsistent and/or conflicting data.

## Conclusions

Variability of practice pattern by maternity centres confirmed to be the major driver of variability of LoS following childbirth in FVG.

Various organizational options are available to contain LoS after CS and reduce avoidable health care cost whilst maintaining and even improving the efficiency and quality of postnatal care. A planned contraction in the number of hospital beds, combined with the implementation of primary care services could contribute to effectively reduce the average LoS and apply policies of ED after CS, as successfully accomplished in some countries. Further in-depth interventions to achieve cost-effective obstetric outcomes could entail limiting the recourse to CS in absence of any clinical indication, changes in the hospital payment system and higher coordination of diagnostic and treatment paths within each maternity unit.

## Data Availability

This study analyzed third party data, extracted from the Regional Repository of Friuli Venezia Giulia (FVG), a database anonymously storing potentially sensitive information. Access to this database is therefore subject to permission from the Regional Health Authority of FVG. Contact: Epidemiology & Health Information Service; Central Health Directorate; Health & Social Integration; Social & Family Policies; Via Pozzuolo 330, 33100, Udine, Italy. Tel: + 39 0432 805661; email: salute@certregione.fvg.it.
